# A bioinformatic framework for immune repertoire diversity profiling enables detection of immunological status

**DOI:** 10.1186/s13073-015-0169-8

**Published:** 2015-05-28

**Authors:** Victor Greiff, Pooja Bhat, Skylar C. Cook, Ulrike Menzel, Wenjing Kang, Sai T. Reddy

**Affiliations:** ETH Zürich, Department of Biosystems Science and Engineering, Basel, 4058 Switzerland

## Abstract

**Background:**

Lymphocyte receptor repertoires are continually shaped throughout the lifetime of an individual in response to environmental and pathogenic exposure. Thus, they may serve as a fingerprint of an individual’s ongoing immunological status (e.g., healthy, infected, vaccinated), with far-reaching implications for immunodiagnostics applications. The advent of high-throughput immune repertoire sequencing now enables the interrogation of immune repertoire diversity in an unprecedented and quantitative manner. However, steadily increasing sequencing depth has revealed that immune repertoires vary greatly among individuals in their composition; correspondingly, it has been reported that there are few shared sequences indicative of immunological status ('public clones'). Disconcertingly, this means that the wealth of information gained from repertoire sequencing remains largely unused for determining the current status of immune responses, thereby hampering the implementation of immune-repertoire-based diagnostics.

**Methods:**

Here, we introduce a bioinformatics repertoire-profiling framework that possesses the advantage of capturing the diversity and distribution of entire immune repertoires, as opposed to singular public clones. The framework relies on Hill-based diversity profiles composed of a continuum of single diversity indices, which enable the quantification of the extent of immunological information contained in immune repertoires.

**Results:**

We coupled diversity profiles with unsupervised (hierarchical clustering) and supervised (support vector machine and feature selection) machine learning approaches in order to correlate patients’ immunological statuses with their B- and T-cell repertoire data. We could predict with high accuracy (greater than or equal to 80 %) a wide range of immunological statuses such as healthy, transplantation recipient, and lymphoid cancer, suggesting as a proof of principle that diversity profiling can recover a large amount of immunodiagnostic fingerprints from immune repertoire data. Our framework is highly scalable as it easily allowed for the analysis of 1000 simulated immune repertoires; this exceeds the size of published immune repertoire datasets by one to two orders of magnitude.

**Conclusions:**

Our framework offers the possibility to advance immune-repertoire-based fingerprinting, which may in the future enable a systems immunogenomics approach for vaccine profiling and the accurate and early detection of disease and infection.

**Electronic supplementary material:**

The online version of this article (doi:10.1186/s13073-015-0169-8) contains supplementary material, which is available to authorized users.

## Background

The lymphocyte repertoire of B and T cells is shaped throughout the lifetime of an individual; in response to environmental and pathogenic antigen challenge, lymphocytes clonally expand and are selected in a highly specific manner. Therefore, the immune receptor clonal diversity and distribution, which summarize the state of clonal selection and expansion, may serve as a fingerprint of an individual’s current immunological status (e.g., healthy, infected, vaccinated), and may thus be exploited for immunodiagnostic applications [1]. There is an enormous diversity of B-cell receptors (BCRs, antibodies) and T-cell receptors (TCRs), theoretically approaching 10^13^ and 10^18^ protein sequences, respectively [2]. Only very recently, through the advent of high-throughput sequencing (HTS), has it become possible to capture the immense clonal diversity and distribution of BCR and TCR repertoires at high resolution [1, 3–5].

While immune repertoire sequencing datasets have steadily increased from 10^3^ to 10^6^ sequencing reads per sample [6–10], it has still remained a challenge to extract from large-scale repertoire data immunological status-specific fingerprints of *entire* repertoires for systems medicine and immunodiagnostics application [1, 11, 12]. In fact, due to both biological and technological reasons, immune repertoire data are quasi-distinct across individuals (humans or mice) with respect to their clonal composition [8]. Clones are predominantly defined based on the complementarity determining region 3 (CDR3) of BCR heavy chains or TCR beta chains [1, 9, 13], which contributes most to the BCR/TCR binding specificity. Biologically, as a result of junctional recombination, P/N nucleotide editing and somatic hypermutation (for BCR/antibodies) [14], the protein sequence space of CDR3s is immense and renders the finding of significant overlap between repertoires highly unlikely. Indeed, BCR-CDR3 sequences from both unimmunized and immunized/vaccinated individuals show small to no sequence overlap [15–17]. Although the incidence of public T-cell clones is higher than that of B cells due to the lack of secondary diversification by somatic hypermutations (SHM), previous HTS studies indicated that their numbers are low compared with the size of the entire T-cell repertoire at any given time (see [8] and references in Robins *et al*. [18]). Technologically, sequencing error, PCR error and limited sequencing depth further decrease the likelihood of discovering 'public' clones [9, 19–21]. In summary, lymphocyte repertoires are quasi-distinct in clonal composition and distribution and this is largely independent of immunological status. This restricts the comparison of immune repertoires across individuals to public clones, thus disregarding a wealth of additional information present in entire immune repertoires, which consequently limits a deeper understanding of lymphocyte repertoires and hampers the development of robust immune-repertoire-based diagnostics.

**Fig. 1:**
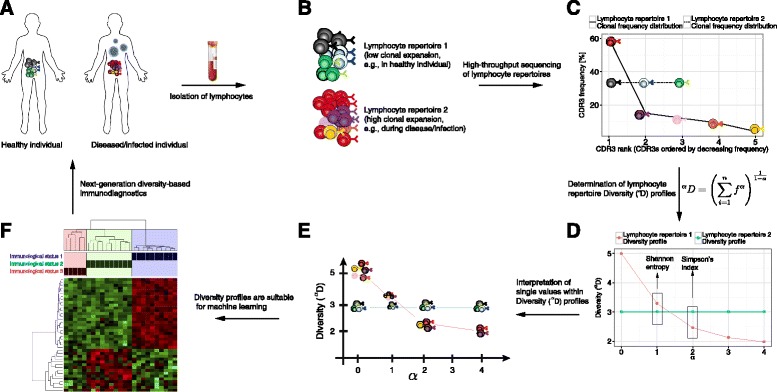
Rendering HTS repertoire data suitable for machine learning-based immunodiagnostics. **a** The clonal distribution and diversity of lymphocyte repertoires may represent a fingerprint of an individual’s current immunological status (e.g., healthy, vaccinated, diseased/infected). **b** Lymphocyte repertoire 1 represents a uniform repertoire (e.g., resembling that of a healthy individual) as opposed to lymphocyte repertoire 2, which shows a large clonal expansion (few clones dominate the repertoire, e.g., as a result of disease/infection or vaccination). Each color describes one lymphocyte clone (usually defined by the CDR3). **c** The immediate output of HTS datasets are immune repertoire clonal frequency distributions, which are composed of the frequency of each clone (where frequency is the proportion of the sequencing reads bearing the same clonal identifier [e.g., CDR3 amino acid sequence]). These distributions differ in clonal composition even in inbred mice [9, 15] (Additional file 4); this renders the application of machine learning approaches highly problematic **(f)** as they require identical composition. **d** Diversity (^α^D, derived from the Rényi entropy) alleviates the problem of incomparable datasets by projecting clonal frequency distributions onto the same (reduced) alpha space. Shannon diversity (alpha = 1) and Simpson’s index (alpha = 2) are widely used for diversity comparisons but, depending on the dataset structure, show qualitatively inconsistent Diversity values (Additional file 2). **e** The Diversity value ^α^D for each alpha signifies an equivalent repertoire in which all clones are equally abundant. These equivalent repertoires represent different portions of the original repertoires, with only the top clones remaining as alpha tends towards infinity. **f** Diversity profiles (vectors of alpha values) are of identical (alpha-)composition and are therefore suitable for cross-repertoire comparisons by machine learning approaches allowing for their potential application in next-generation immunodiagnostics

The challenges in comparing immune repertoires in their entirety led to the adoption of sequence-independent quantifiers of clonal diversity (also termed 'diversity indices') [7, 22–25]. These quantifiers offer the possibility to correlate immune repertoire *diversity* to immunological status and in doing so readily allow for immune-repertoire-based comparisons across individuals [7, 22, 26–30]. It has been long known that there is a continuum of possible diversity measures all of which are related to Rényi’s definition of generalized entropy [31, 32]. However, the extent to which diversity indices reliably capture the status-specific information of immune repertoires still remains an area in need of deeper investigation. The premise that immune repertoires accurately reflect immunological status serves as the basis for the alluring possibility that diversity analysis could be exploited for applications such as next-generation immunodiagnostics, which may in the long term enable the early detection and diagnosis of disease/infection and provide more quantitative vaccine profiling [4, 7, 33, 34]. Disconcertingly, it has been noted that single diversity indices, such as the Shannon or Simpson’s diversity index, can yield qualitatively different results [35–37] (Fig. 1c); this finding raised questions regarding the consistency of immunological classification based on single diversity indices [22, 24, 26, 30]. Therefore, we set out to answer the following questions: (i) To what extent do diversity indices robustly capture the immunological information inherent in high-throughput immune repertoire sequencing data? (ii) How can diversity indices be used to quantitatively define and reveal immunological status?

To answer these questions, we have developed a sequence-independent and highly scalable bioinformatical framework for the analysis of immune repertoire diversity (Fig. 1). The framework relies on the transformation of clonal frequency distributions into alpha-parameterized profiles of Hill-based diversities (^alpha^*D*), resulting in a continuum of diversity indices with an alpha-modulated sensitivity for the relatively rare clones in a lymphocyte repertoire. As opposed to single diversity values, our profile-based framework enabled the reliable capture of immune repertoire clonal frequency distributions, and thus, the majority of potential immunological information coded therein. Leveraging machine learning approaches we could show that clonal frequency distributions hold indeed a potentially large amount of immunological fingerprint information, since we could classify with high accuracy (≥80 %) BCR- and TCR-repertoire datasets derived from a wide range of immunological statuses such as healthy, transplantation and lymphoid cancer. Thereby, our work represents a proof of principle for the successful utilization of immunogenomic lymphocyte repertoire fingerprinting and the development of diversity-based immunodiagnostics for early detection of disease and infection [4, 5, 20, 38].

## Methods

### Experimental datasets

We compiled four experimental datasets, which are characterized below. Sequencing read statistics are summarized in Additional file 1. Further quality and read information on datasets 1–3 can be found in the respective publications.

#### Dataset 1

Human T-cell origin: HTS data were published by Muraro and colleagues [10] as part of a phase II trial for poor-prognosis multiple sclerosis. TCR-Vβ gene sequencing was performed to assess both CD4 and CD8 repertoires per individual before and at two time points, 2 months and 12 months, after autologous hematopoietic stem cell transplantation in a cohort of 24 patients. The preprocessed CDR3 clonotype frequency distributions were downloaded from [39].

#### Dataset 2

Human B-cell origin: HTS BCR data were published by Bashford-Rogers and colleagues [7]: peripheral blood mononuclear cells (PBMCs) were isolated from 10 ml of whole blood from 13 healthy volunteers and 11 patients with chronic lymphocytic leukemia (CLL) and immunoglobulin heavy chains were amplified. HTS was performed on the VH gene using Roche 454. The raw data were downloaded (European Nucleotide Archive accession number ERP002120) and submitted to ImMunoGeneTics (IMGT)/HighV-QUEST [40] for CDR3 annotation. IMGT-annotated data were preprocessed analogously to that of dataset 4.

#### Dataset 3

Human B-cell origin: HTS BCR data published by Jackson and colleagues [16]. PBMCs were isolated from blood drawn from 14 individuals before and on days 7 and 21 after influenza vaccination. BCR-VH genes were PCR amplified, sequencing was performed using Roche 454. The raw data were downloaded (dbGaP accession number phs000760.v.1.p1) and submitted to IMGT for CDR3 annotation. IMGT-annotated data were preprocessed analogously to that of dataset 4.

#### Dataset 4

Murine B-cell origin: From a single mouse immunized and boosted with one chicken gamma globulin (CGG) conjugated to 4-hydroxy-3-nitrophenylacetyl (NP; NP-CGG), naïve follicular B cells (NFBCs, IgM), antibody-secreting cells (ASCs, IgM/IgG) and plasma cells (PCs, IgM/IgG) were isolated using fluorescence-activated cell sorting (FACS). The experimental workflow and data preprocessing are described below.

##### Animal experiments and cell isolation

All animal experiments were performed under the guidelines and protocols approved by the Basel-Stadt cantonal veterinary office (Basel-Stadt Kantonales Veterinäramt Tierversuchsbewilligung #2582). One BALB/c mouse (Charles Rivers Laboratories, 8–10 weeks old), housed under specific pathogen-free conditions and maintained on a standard chow diet, was immunized with 50 μg alum-precipitated CGG conjugated to NP (NP-CGG, BioCat) and boosted with 50 μg NP-CGG in phosphate-buffered saline three weeks later. The mouse was sacrificed 7 days after the boost. The spleen was harvested and ≈ 3 Mio splenic NFBCs (CD19^+^/CD138^−^/IgM^+^/IgD^++^/CD23^++^/CD21^+^/GL7^−^), ≈200,000 splenic ASCs (CD19^int^/CD138^++^/MHCII^hi+lo^/CD38^+^/CD93^−^/GL7^−^), and 5000 splenic PCs (CD19^−^/CD138^++^/MHCII^lo^/CD38^+^/CD93^−^/GL7^−^) were FACS-isolated and sorted into Trizol.

##### Preparation of antibody libraries for high-throughput sequencing

Total RNA extraction and antibody library generation were performed from the above described three cell populations (NFBCs, ASCs, PCs) using a primer extension method as described previously [41]. NFBCs were amplified with an IgM-specific (in italics) reverse primer (GAGGAGAGAGAGAGAG *CGAGGGGGAAGACATTTGGG*) containing the overhang region as previously described [41] while the ASC and PC samples were amplified using a mix of IgM (GAGGAGAGAGAGAGAG CGTGAT*CGAGGGGGAAGACATTTGGG*)- and IgG (GAGGAGAGAGAGAGAG ACATCG*CCARKGGATAGACHGATGGG*)-specific reverse primers containing an identification tag (underlined region) within the primer sequence that was later used in the data pre-processing for discrimination between isotypes from each Illumina barcoded sample.

##### Illumina sequencing and data preprocessing

All samples were sequenced using the Illumina MiSeq platform with 2 × 250 bp paired-end reads with high mean quality Phred scores ranging from 35 to 36 and ≈ 90 % of bases having a quality Phred score of >30. Raw data can be accessed from zenodo [42]. Forward and reverse reads were paired using PANDAseq (v.2.7, threshold parameter t = 0.6) [43]. The PANDAseq pairing efficiency was >97 %. The splitting of the PANDAseq fasta files into IgM and IgG sequencing reads was performed using the function vcountPDict (allowing indels and a maximum number of five mismatches) from the R package ShortRead [44] with a ≈ 95 % efficiency. Full-length VDJ region annotation of successfully paired sequences was performed using IMGT/HighV-QUEST [45, 46]. For downstream analyses, sequences were pre-processed and reads were only retained if answering the following requirements: (i) the IMGT-indicated 'Functionality' of the sequencing was 'productive'; (ii) CDR3s were of minimal length of four amino acids; (iii) CDR3s were present with a minimum abundance of 2. For all analyses, CDR3 abundances were calculated based on occurrence of exact amino acid sequences (100 % identity).

### Simulation of Zipfian distributions

Zipfian distributions were simulated using the Zipf-Mandelbrot law implemented in the zipfR R package [47]. The respective probability density function used for simulations is given by

$$ g\left(\pi \right):=\left\{\begin{array}{r}\hfill C\cdot {\pi}^{\mathrm{Zipf}\hbox{-} \upalpha +1},\\ {}\hfill 0,\end{array}\kern1em \begin{array}{l}\left|0\le \uppi \le \mathrm{Zipf}-B\right.\hfill \\ {}\left|\mathrm{otherwise}\right.\hfill \end{array}\right. $$

Here, Zipf-α ∈ (0, 1) and Zipf-B ∈ (0, 1) are two free parameters. C is a normalizing constant. B corresponds to the probability π_1_ of the most frequent species (clone) [48].

### Diversity profiles

Clonal diversity was defined as

$$ {}^{\upalpha}\mathrm{D}\left(\mathrm{f}\right)={\left({\displaystyle {\sum}_{\mathrm{i}=1}^{\mathrm{n}}{\mathrm{f}}_{\mathrm{i}}^{\upalpha}}\right)}^{\frac{1}{1\hbox{-} \upalpha}}, $$

where *f* is the clonal frequency distribution with *f*_*i*_ being the frequency of each clone and *n* the total number of clones [31, 32, 49]. The α-values represent weights, which means as α increases, higher frequency clones are weighted more. The alpha-parameterized Diversity creates for a given array of alphas a diversity index *profile* (short: diversity profile or $\overrightarrow{{}^{\alpha }D}$). Diversity is not defined for the case alpha = 1. However, we used L’Hospital’s rule to find that as alpha tends to 1, Diversity tends to the Shannon entropy. Thus, the Shannon entropy is a special case of the Diversity for alpha = 1.

Evenness (^α^E) describes the extent to which a given species frequency vector is distanced from the uniform distribution species frequency vector and is defined as

$$ {}^{\upalpha}\mathrm{D}=\mathrm{S}\mathrm{R}{\times}^{\upalpha}\mathrm{E} $$

where SR is the species richness (SR = ^α=0^*D*), that is, the number of unique clones in a repertoire dataset.

Diversity and Evenness profiles were calculated in a range of alpha = 0 to alpha = 10 with a step size of 0.2 if not specified otherwise. The alpha range was chosen based on the observation that most profiles leveled off toward an alpha of 10 (Additional file 2).

### Hierarchical clustering

Using Euclidean distance as a distance metric, clustering of profiles was performed using the 'complete linkage' clustering algorithm performed by the R function hclust() from the stats R package [50]. Hierarchical clustering was visualized by dendrograms using the ggdendro R package [51] and heatmaps using the gplots [52], NMF [53] and heatmap.plus [54] R packages. For Evenness profiles, we performed the above mentioned clustering algorithm on the correlation matrix in order to obtain scale invariance and focus exclusively on differences of shapes in Evenness profiles (for Diversity profiles the focus lay on shape and magnitude differences).

The correlation between dendrograms was determined using the cor_cophenetic() function from the dendextend R package [55]. Specifically, it calculates the correlation between any two cophenetic distance matrices of two given hierarchical clustering trees (dendrograms). The cophenetic distance between two observations that have been clustered is defined to be the intergroup dissimilarity at which the two observations are first combined into a single cluster. The values given by the cor_cophenetic() function range between −1 and 1. Values near zero signify that two trees were not statistically similar [56].

### Support vector machine analysis and feature selection

Support vector machine (SVM) analysis was performed using the Potential Support Vector Machine (P-SVM) [57], which combines linear classification (classification of immunological status) of Diversity and Evenness profiles with the selection of a minimal subset of alpha values achieving the highest prediction accuracy (feature selection). The goal criterion of classification performance was balanced prediction accuracy (BACC = (Sensitivity + Specificity)/2). The classification performance was measured using nested leave-one-out cross-validation, where feature selection and hyperparameter selection were performed in the inner cross-validation loop independently of the test sample of the outer cross-validation loop (Additional file 3). The inner loop was used to determine the combination of parameters allowing the best classification performance: the cost parameter c was varied from 1 to 17 in five equally spaced steps and the regularization parameter ε was chosen as 2^i^ with i = −3,−2,…,3, 4. In order to obtain compact models that only use a small set of features, all parameter combinations in the inner cross-validation loop for which more than three models exceeded an upper limit of 20 selected alpha values were excluded. A flowchart of the P-SVM algorithm is given in Additional file 3.

Correlations between P-SVM-selected alpha values and immunological status may occur by chance. In order to exclude such random effects, permutation testing consisting of 1000 independent random shuffles of the label vector and subsequent determination of BACC was performed. BACCs were regarded as significant if the number of BACCs of the shuffled label vectors exceeding that of the original label vector was lower than 10 out of 1000 (*p* < 0.01) [58].

## Results

### Diversity profiles comprehensively characterize immune repertoire structure

To characterize immune repertoire clonal structure, we employed the Hill-based Diversity (hereafter referred to as “Diversity”), which is based on Rényi’s definition of generalized entropy [31, 32, 49], ${}^{\upalpha}\mathrm{D}\left(\mathrm{f}\right)={\left({\displaystyle {\sum}_{\mathrm{i}=1}^{\mathrm{n}}{\mathrm{f}}_{\mathrm{i}}^{\upalpha}}\right)}^{\frac{1}{1\hbox{-} \upalpha}}$, where *f* is the clonal frequency distribution with *f*_*i*_ the frequency of each clone and *n* the total number of clones. Currently, the structure of BCR and TCR repertoires is represented by their clonal frequency distributions as it summarizes the state of clonal expansion and selection (Fig. 1c). Notably, our framework is independent of specific definitions of clonality (e.g., unique CDR3 sequences). The alpha-parameterized Diversity unifies many previously established diversity indices (SR, ^α=0^*D* [9, 59]; Shannon,^α=1^*D* [24, 60]; Simpson’s, ^α=2^*D* [7, 61]; Berger-Parker ^*α* → ∞^*D* [9, 62]) and creates, for a given array of alphas, a diversity index *profile* (diversity profile in short or $\overrightarrow{{}^{\alpha }D}$). The Diversity represents the number of equally common species (e.g., clones) required to yield a particular value of ^α^*D* [49, 63] (Fig. 1e). The α-values represent weights, which means as α increases, higher frequency clones are weighted more (Fig. 1e). While the lower limit of alpha tends to the SR (SR = ^α=0^D = n), the upper limit of alpha is dominated by the frequency of the most abundant clone of the respective repertoire (^*α* → ∞^*D* = −log sup *f*_*i*_). Since each alpha value focuses on a different stretch of the immune repertoire (Fig. 1d, e), the Diversity forms a continuum of viewpoints on the same underlying immune repertoire structure.

**Fig. 2:**
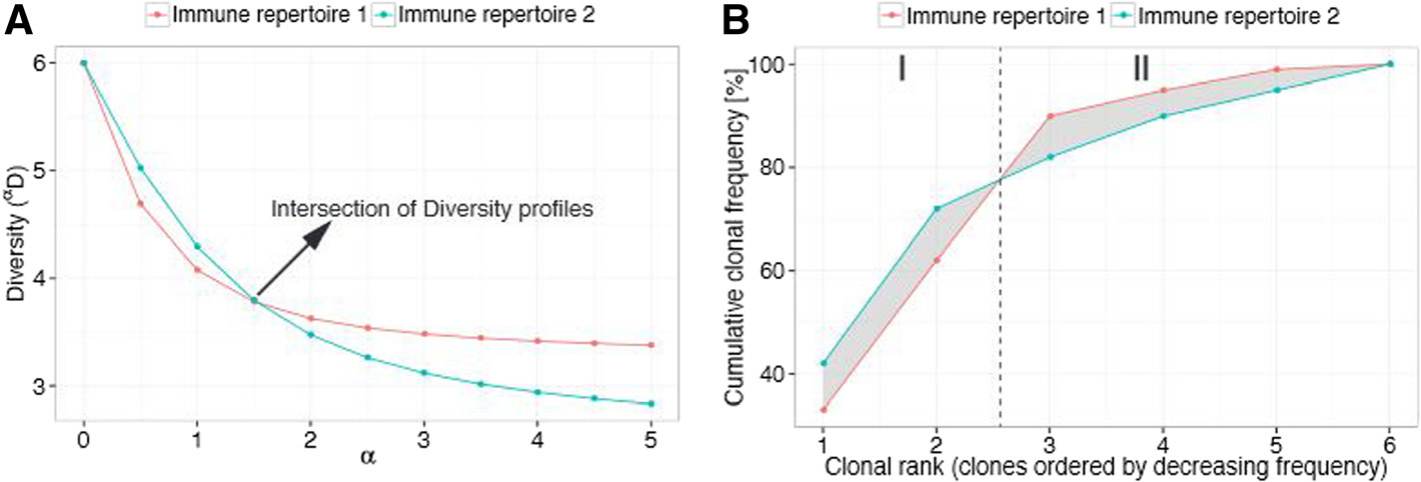
Diversity profile intersection predicts differential sub-repertoire clonal expansion. **a** Intersecting Diversity (^α^ *D*) profiles of two immune repertoires with different clonal frequency distributions are shown (immune repertoire 1 with clonal frequencies of 33 %, 29 %, 28 %, 5 %, 4 %, 1 %; immune repertoire 2 with clonal frequencies of 42 %, 30 %, 10 %, 8 %, 5 %, 5 %). **b** Intersection of frequency-ordered cumulative frequency distributions of immune repertoires shown in **(a)**. The Diversity (^α^ *D*) function is Schur-concave, which predicts intersection of cumulative frequency curves if intersection in the profile space has occurred. Since cumulative frequency curves were derived from frequency-ordered clonal frequency distributions, the exact delineation of differentially expanded sub-repertoires becomes possible. Here, until clonal rank 2 immune repertoire 2 is higher clonally expanded (*area I*) whereas the opposite is true from clonal rank 3 onward (*area II*). The grey-shaded area indicates the clonal expansion difference between the two immune repertoires. Since the difference in clonal expansion is expressed in percent, the determination of relative oligo-/polyclonality with respect to a given region of the immune repertoires becomes possible

Recent reports have provided evidence that immune repertoires follow a power law distribution, more specifically, Zipf-like distributions [64] (linear correlation between log(clonal frequencies, f) and log(clonal rank)). Our own and previously published data from others [7, 10, 16] are in agreement with these findings both for B- and T-cell repertoires (Additional file 4) [41]. The influence of alpha on Zipf-like clonal frequency distributions is high, in contrast to uniform ones (Fig. 1d, e), and may lead to the intersection of Diversity profiles (Fig. 1d, e; Additional file 2). Naturally, this renders qualitative diversity comparisons based on single diversity indices questionable; a diversity index before the intersection may reveal that one repertoire is more diverse than the other while the reverse is true for an index with a different alpha value after the intersection (Fig. 1d, e; Additional file 2). Indeed, we found diversity profile intersection for all of the BCR and TCR datasets within and across immunological status, which were as varied as healthy, cancer (CLL), influenza vaccination and transplantation (Additional file 2). Therefore, we set out to attribute an immunological meaning to diversity profile intersection by connecting diversity profiles directly to the underlying immune repertoire (Fig. 2). In order to accomplish this we took advantage of the Schur-concavity of the Diversity functions. Briefly, the intersection of Schur-concave functions (Fig. 2a) predicts a likewise intersection of the underlying rank-ordered *cumulative* frequency distributions (Fig. 2b) [35]. Thus, the intersection of diversity profiles indicates that the underlying clonal frequency distributions differ markedly in their shape on *several* (at least two) clonal regions of the repertoire (Fig. 2b), indicating the existence of *qualitatively varying* clonal expansion differences between immune repertoires (Fig. 2b). By virtue of the linkage of diversity profiles and underlying frequency distributions, it is now possible to predict these differences in clonal expansion only based on the respective diversity profiles. Of note, without the use of profiles, the differences in clonal expansion may have remained undetected. Immunologically, the intersection of diversity profiles may be explained by different underlying kinetics of clonal expansion: while one repertoire is already highly expanded possibly due to an acute infection (showing a minority of clones with higher frequency and a majority of clones with very low diversity), the other repertoire could be more evenly distributed with most of the clones being of similar frequency as this may be reflective of an antigen-inexperienced cell population.

**Fig. 3:**
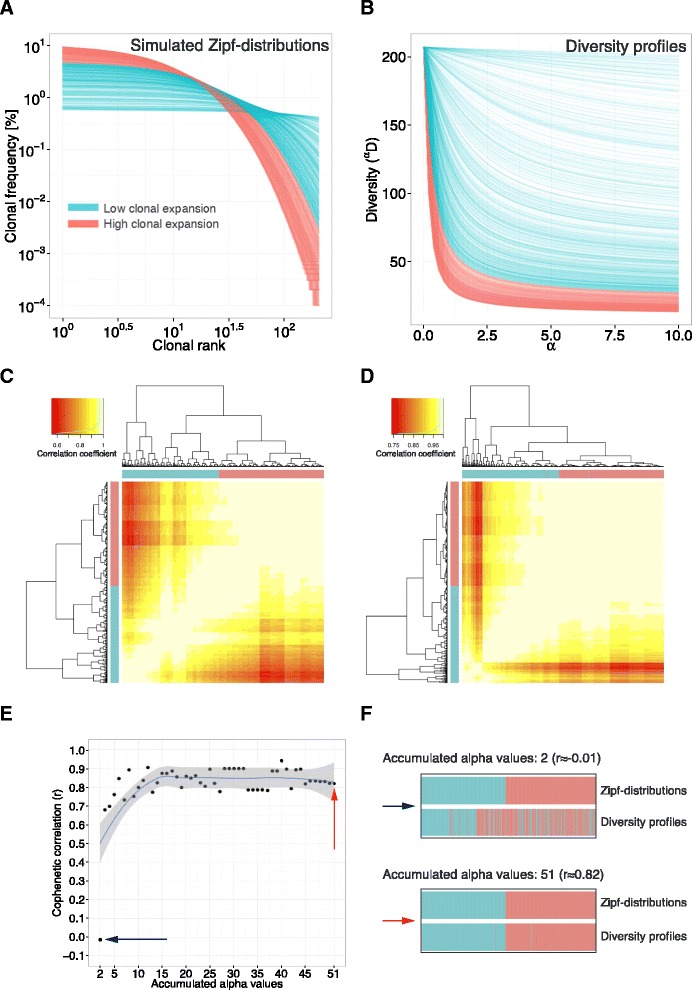
Diversity profiles recover the underlying frequency distribution to a large extent. **a** Simulation of 1000 clonal frequency (Zipf) distributions of varying degree of clonal expansion (Zipf-alpha = 0.1, Zipf-B ∈ [0.001, 0.1]), but equal clonal composition. Distributions were colored by extent of clonal expansion (blue, low clonal expansion; red, high clonal expansion). **b** Diversity profiles of Zipf-distributions **(a)** were plotted for alpha values ranging from 0 to 10. Diversity profiles were colored by the respective Zipf-distribution. **c** Zipf-distributions **(a)** were hierarchically clustered based on Pearson correlation distance in order to only take into account the shape of the distributions. Hierarchical clustering was visualized using heatmaps, in which each tile represents the Pearson correlation coefficient between any two distributions. Row and column color (blue, red) bars indicate the respective degree of clonal expansion of each distribution as shown in **(a)**. **d** Diversity profiles of Zipf-distributions **(a)** were hierarchically clustered based on Pearson correlation distance in order to only take into account relative clonal expansion differences. **e** The cophenetic correlation of the dendrograms of Zipf-distributions **(c)** and of Diversity profiles **(d)** was determined as a function of a growing [accumulating] number of alpha-values used — the number of alpha values was varied between 2 and 51 within an alpha range of 0 to 10 (step size of 0.2). The cophenetic correlation (r) between dendrograms of frequency distributions and Diversity profiles increases with increasing number of alpha values used reaching r ≈ 0.94 for 40 and r ≈ 0.82 for 51 alpha values used. **f** Color bars as used in heatmaps in **(c)** and **(d)** are shown to visualize the correspondence of clustering of Zipf distributions and Diversity profiles for the two extreme cases of the number of alpha values used: 2 (blue arrow) and 51 (red arrow)

Having *linked* diversity profiles to frequency distributions, we next went one step further to quantitatively test how sensitively diversity profiles *represent* the underlying clonal frequency distribution. Only when the representation is of high confidence is the maximum amount of immunological information inherent to the clonal frequency distribution captured by the considerably lower-dimensional diversity profile. To test the level of confidence of diversity profiles, we hierarchically clustered 1000 in silico generated Zipf frequency distributions representing various states of repertoire clonal expansion (Fig. 3a, c) as well as their corresponding Diversity profiles (Fig. 3b, d). The number of our simulated Zipf distributions exceeds the size of published immune repertoire HTS datasets by one to two orders of magnitude [7, 10, 16]. The Zipf-distributions were of identical dimension and composition, which allowed for their hierarchical clustering. The hierarchical clustering dendrograms of Zipf distributions (Fig. 3c) and Diversity profiles of 51 alpha values in a range of 0 to 10 (Fig. 3d) reached a cophenetic correlation of r ≈ 0.82 (Fig. 3e), which reflects a highly faithful representation of immune repertoire structure by diversity profiles. The cophenetic correlation coefficient measures the similarity between dendrograms (see *Methods*). Next, we proceeded to determine the dependence of the correlation of distribution and diversity profile clustering on the number of alpha values used. We found that the positive correlation between both dendrograms levels off towards 15 alpha values (r ≈ 0.82); the maximum correlation of r ≈ 0.94 is reached with 40 alpha values (Fig. 3e). These simulations suggest that diversity *profiles* reflect with higher accuracy the clonal distributions of immune repertoires when compared with *single diversity measures*. Indeed, low numbers (<5) of alpha values yielded correlations below r = 0.8 (Fig. 3e); profiles composed of two alpha values, which is in the range of the commonly used Shannon and Simpson’s index, did not recover the hierarchical clustering of the simulated Zipf-distributions (r ≈ 0) (Fig. 3f).

Although Diversity profiles accurately *reflect* immune repertoire clonal (Zipf) frequency distributions, they are unfit to *quantify* their degree of clonal expansion. Therefore, we showed using the Rényi divergence [31] that the Diversity is divisible into two components: SR (SR = ^α=0^*D*) and Evenness (^α^E: ^α^D = SR × ^α^E) (Additional file 5). Evenness describes the extent to which a given species frequency vector is distanced from the uniform distribution species frequency vector, and is thereby immunologically interpretable as the *extent of clonal expansion* of a given immune repertoire. Diversity and Evenness are mathematically independent [63], signifying the inability to infer Evenness solely based on Diversity and vice versa. Thus, Evenness and Diversity are two independent descriptors of lymphocyte clonal frequency distribution. Since Evenness profiles are SR-scaled versions of diversity profiles, it follows that they also represent immune repertoire frequency distributions to a high degree (Fig. 3).

While HTS has enabled an unprecedented depth of coverage of immune repertoires (i.e., 10^5^–10^6^ sequencing reads per repertoire), there is still a vast undersampling of biological diversity, especially in human samples [9, 65]. Consequently, we investigated the robustness of Diversity and Evenness profiles to varying sequencing depth (technological undersampling). To this end, we simulated Zipf distributions of a wide range of states of clonal expansion using 10^6^ reads (Additional files 2A and 4A) as this represents the magnitude of reads reported using current HTS instruments (e.g., Illumina) [7, 24, 41]. Across various sequencing read depths (10 to 100 %), we determined both the pairwise probability of the intersection of repertoires (qualitative robustness; Additional files 6A and 7A) and the mean distance between profiles (quantitative robustness; Additional files 6B and 7B). We found that Diversity profiles were qualitatively and quantitatively robust across the entire sampling range (from 10 % sampling onward; Additional file 6) whereas in case of qualitative robustness, Evenness had to rely on higher percentages of reads (>90 %) to reach robustness to technological undersampling (Additional file 7) [9].

Thus, Diversity and Evenness profiles reliably conserve the information of higher dimensional frequency distributions (Fig. 3; Additional files 6 and 7) and reflect accurately the state of clonal expansion (Fig. 2; Additional file 2). Importantly, immune repertoires, across individuals and across time points within individuals, differ in clonal composition, and thus are unsuitable for machine learning analyses, which require the compared repertoires to be of identical composition. Diversity and Evenness profiles, however, fulfill *by construction* this requirement (Fig. 1c) and therefore enabled us to perform cross-individual comparison of *entire* immune repertoires.

### Diversity and Evenness profiles can predict the immunological status of immune repertoires: a proof of principle for a repertoire-based immunodiagnostics pipeline

As environmental and pathogenic exposure greatly influence clonal frequency distributions, diversity profiles $\left(\overrightarrow{{}^{\alpha }D},\overrightarrow{{}^{\alpha }E}\right)$ may be reflective of an individual’s current immunological status [66] (Fig. 3). To test this, we applied our profile-based framework and machine learning to in-house and publicly available experimental HTS data of both BCR/antibody variable heavy chain (VH) and TCR variable beta chain (Vβ) repertoires in various human and murine lymphocyte populations. We compiled four datasets using the CDR3 as clonal identifier. Dataset 1 consists of HTS data of sorted CD4 and CD8 T cells, which was part of a phase II trial for poor-prognosis multiple sclerosis [10]. Sequencing was performed on the level of TCR Vβ to assess the repertoires before (baseline, 24 samples for both CD4/CD8) and at two time points (2 and 12 months, 24 samples each for both CD4/CD8) after autologous hematopoietic stem cell transplantation. Dataset 2 consists of HTS of VH from B cells obtained from peripheral blood of healthy volunteers (13 samples) and patients with CLL (11 samples) [7]. Dataset 3 is composed of human HTS of VH from peripheral blood B cells of 14 individuals prior to and 7 and 21 days after seasonal influenza vaccination [16]. Dataset 4 is composed of HTS of VH from murine NFBCs and antibody-secreting B cells.

**Fig. 4:**
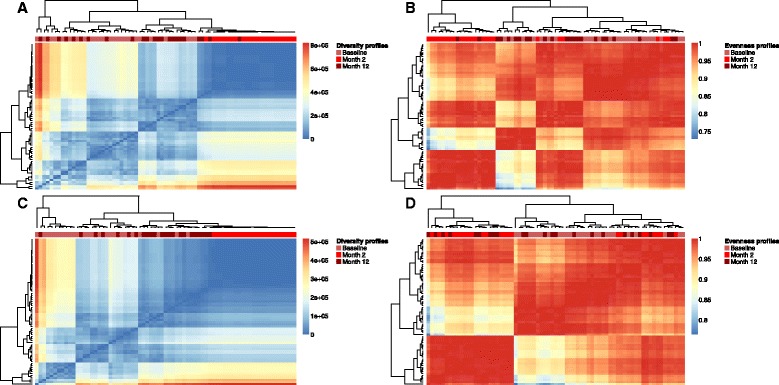
Diversity and Evenness profiles resolve stages of hematopoietic stem cell transplantation. **a–d** Hierarchical clustering was performed based on Euclidean distance for Diversity profiles and correlation-based distance for Evenness profiles of dataset 1 and visualized using heatmaps. The heatmaps depict the pairwise distances/Pearson correlation coefficients of all profiles determined (see *Methods* for further details). Both for CD4 and CD8 T-cell repertoires, Diversity **(a, c)** and Evenness **(b, d)** profiles from '*Month 2*' (*blue*) after transplantation cluster together as do profiles of '*Baseline*' measurements (*green*) and '*Month 12*' (*red*) after transplantation (*red color bar*). Of note, for CD8 datasets, Diversity profiles cluster almost perfectly by each of the three statuses (Baseline, Month 2, Month 12). Diversity and Evenness profiles were calculated in a range of alpha = 0 to alpha = 10 with a step size of 0.2. Sample numbers: 24 per immunological status and T-cell population

**Fig. 5:**
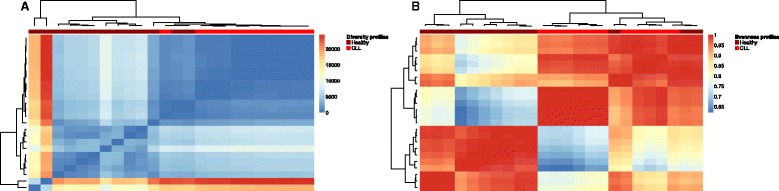
Diversity and Evenness profiles separate healthy from cancer-afflicted individuals. **a, b** Analogously to Fig. 4, Diversity **(a)** and Evenness **(b)** profiles of dataset 2 were hierarchically clustered. Diversity and Evenness profiles separate healthy and CLL-afflicted individuals well (*red color bar*). Diversity and Evenness profiles were calculated in a range of alpha = 0 to alpha = 10 with a step size of 0.2. Sample numbers: healthy, 13; CLL, 11

In order to visualize possible immunological phenotypic differences of Diversity and Evenness profiles, we used hierarchical clustering. Diversity profiles were clustered by Euclidian distance to take into account the SR differences between repertoires, whereas Evenness profiles were clustered by correlation distance in order to exclusively focus on their shape (relative degree of clonal expansion). For dataset 1 (human, TCR-Vβ, baseline versus transplantation), we found that both Diversity and Evenness profiles cluster by 2 months, and baseline with 12 months, which is in line with the intuition that 12 months after hematopoietic stem cell transplantation the immune system has recovered the pre-surgery baseline state whereas 2 months after transplantation the T-cell repertoire has assumed a perturbed state (Fig. 4a–d). For dataset 2 (human, BCR, healthy versus CLL), we found that Diversity and Evenness profiles cluster samples of B-cell repertoires of healthy and CLL-afflicted patients well (Fig. 5a, b).

For dataset 3 (human, BCR, baseline versus influenza vaccination), we found that profiles did not cluster by immunological status. Interestingly, diversity profiles of our two human BCR/antibody datasets 2 and 3 clustered apart from one another well (Additional file 8). However, healthy individuals across both datasets did not cluster together, suggesting that dataset clustering may still be heavily influenced by non-biological factors such as laboratory origin, sample preparation, and sequencing instrument. Finally, for dataset 4 (murine, BCR, naïve versus antibody secreting cells), naïve B cells distinctly clustered apart from repertoires of antibody-secreting cells (Additional file 9). We also determined that the robustness of profile clustering to varying sequencing depth was high: we in silico generated 20 Zipf-distributions of 10^6^ reads and assessed whether clustering was consistent between 10 % and 100 % of sequencing reads. Indeed, we found that both Diversity and Evenness profile clustering was robust starting from 30 % of the total data (Additional file 10).

Finally, in order to quantify the immunological predictive performance of Diversity and Evenness profiles, we applied SVM analysis and feature selection (Additional file 3) to dataset 1 and 2 profiles (Table 1) since they showed immunological status-dependent profile differences by unsupervised hierarchical clustering (Figs. 4 and 5). The prediction accuracy of our SVM analysis, defined as the mean of prediction specificity and sensitivity, ranged between ≈ 80 % and ≈ 95 %, showing that diversity profiles correlated with immunological status. SVM feature selection determined a median number of 2 to 11 alpha values as optimal for reaching the highest prediction accuracy (Table 1). Restricting SVM analyses to conventional *single* Diversity and Evenness indices ^α=1,2^*D* and ^α=1,2^*E* (representing the [scaled] exponential of the Shannon entropy and Simpson’s index, respectively) resulted in a drop of prediction accuracy by ≈ 5 % to ≈ 30 % (Additional files 11 and 12). In addition, for dataset 2 (healthy versus CLL), all (2 out of 2), and for dataset 1, several (2 out of 4 for Shannon and 1 out of 4 for Simpson’s index) of the single diversity indices-based SVM analyses did not reach significance (p ≥ 0.01), whereas profile-based SVM analyses always yielded (6 out of 6 SVM analyses) p-values of 0 (Table 1; Additional files 11 and 12). This further underlined the advantage of using Diversity and Evenness *profiles* in a prospective immunodiagnostics pipeline (Fig. 1) for a more accurate and robust capture of the immunological status of immune repertoires.

**Table 1:** Diversity (^α^ *D*) and Evenness (^α^ *E*) profiles classify TCR (dataset 1) and BCR (dataset 2) immune repertoires by immunological status with high accuracy

	BACC (%)	Sensitivity (%)	Specificity (%)	Significance (*p*-value)	Median number of alpha-values used
**Dataset 1**					
CD4-Diversity $\left(\overrightarrow{{}^{\alpha }D}\right)$: Month 2 versus Baseline + Month 12	86.5	72.9	100	0	8
CD4-Evenness $\left(\overrightarrow{{}^{\alpha }E}\right)$: Month 2 versus Baseline + Month 12	91.7	83.3	100	0	11
CD8-Diversity $\left(\overrightarrow{{}^{\alpha }D}\right)$: Month 2 versus Baseline + Month 12	79.2	58.3	100	0	9
CD8-Evenness $\left(\overrightarrow{{}^{\alpha }E}\right)$: Month 2 versus Baseline + Month 12	96.9	93.8	100	0	6
**Dataset 2**					
Diversity: Healthy versus CLL $\left(\overrightarrow{{}^{\alpha }D}\right)$	88	77	100	0	5
Evenness: Healthy versus CLL $\left(\overrightarrow{{}^{\alpha }E}\right)$	84	77	91	0	2

The median number of alpha-values employed to reach optimal prediction accuracy (BACC) ranged between 2 and 11. BACCs were computed using nested leave-one-out cross-validation and were regarded as significant if *p* < 0.01. BACC ((Sensitivity + Specificity)/2), balanced prediction accuracy. Diversity and Evenness profiles were calculated in a range of alpha = 0 to alpha = 10 with a step size of 0.2

## Discussion

The non-uniform composition of immune repertoires precludes their comparison using machine learning approaches and consequently the extraction of whole-repertoire immunological fingerprints. Translation into Diversity and Evenness profiles, in contrast, offers the unique advantage to conserve (and potentially extract) the biological information of entire immune repertoire datasets while simultaneously compressing them to a uniform composition. Having shown that immune repertoire frequency distributions are faithfully captured by diversity profiles (Figs. 2 and 3), we applied hierarchical clustering, SVMs and feature selection to diversity profiles and showed that they accurately predict immunological status (Figs. 4 and 5, Table 1). This indicated that the clonal frequency distributions of the datasets analyzed contained immunological information that was consistent across individuals of identical immunological status but differed from those individuals of different status. Therefore, diversity profiles offer a sequence-*independent* approach to immune repertoire-based diagnostics, taking into account the entire clonal structure of the completely sequenced repertoire and not just that of a vanishingly small percentage of potentially shared public clones. SVM analyses revealed that, as opposed to diversity profiles, *single* diversity values were unreliable predictors; for alpha = 1, 4 out of 6, and, for alpha = 2, 3 out of 6 prediction accuracies were insignificant (*p* ≥ 0.01; Additional files 11 and 12). Our diversity *profile*-based approach was highly and consistently reliable as profiles yielded the lowest possible *p*-value for all six prediction scenarios (Table 1). The feature selection of alpha values for prediction of immunological status avoided overfitting. The number of alpha values used is a function of the extent of difference between immunological statuses: the lower the difference, the more alphas will be needed by the SVM (or any other feature selection algorithm) to reach optimal prediction accuracy. For the development of an immunodiagnostics pipeline, the dependence of antibody repertoire structure on demographic factors (age, gender, medical history) deserves further consideration [29, 67].

While we have provided evidence that clonal frequency distributions contain immunologically relevant information, an equally important question is how this information is coded. Clearly, a non-uniform distribution is required for storage of biologically specific information. Indeed, we found, in accordance with previous publications [64, 68], that all datasets analyzed followed a non-uniform, power-law distribution (Zipf-distribution). It has been suggested that Zipf-distributions can naturally arise in response to antigen exposure [66] — modulated by antigenic complexity, exposure frequency and evolutionary optimization [27, 69]. This may explain diversity profile patterns shared across individuals (Figs. 3, 4 and 5). By virtue of the developed bioinformatical framework and high-throughput repertoire sequencing, we are now in the position to investigate whether any given immunological status gives rise to a specific clonal frequency distribution (immunosignature) [9, 70, 71]. Performing these investigations depends on further experimental, technological and statistical advancements.

Experimentally, (single-cell) sorting and sequencing of specific lymphocyte populations (e.g., PCs, memory B cells, effector memory T cells) may increase the antigen-specific signal in diversity profiles by eliminating the noise of non-significant cell populations [3, 15] as well as normalizing the influence of differences in clonality and RNA expression and correcting for PCR and sequencing related biases. This may be especially relevant in PBMC-based repertoire analyses where it remains to be seen whether the clonal expansion signature is dominant enough to show through the background diversity of other cell populations. Thus, a high sequencing depth might also be needed if performing sequencing on bulk unsorted cell populations with a high amount of non-specific signal (Additional file 8) in order to be able to detect immunological status-specific differences.

Technologically, standardization [72, 73] will be needed to increase comparability of repertoire datasets originating from different sources (Additional file 8; batch variance) as currently different experimental approaches (single cell sequencing [74] and heavy/light-chain-pairing [75], unique molecular identifier tagging [76]) and varying computational workflows (quality pre-processing [77], error correction by consensus read formation [21, 78], replicate sequencing [9, 68, 79]) are used for data generation and analysis. Indeed, our results suggested that diversity profile-based analyses were very sensitive to laboratory-dependent experimental workflows (Additional files 1, 8 and 10). For example, assuming that healthy individuals should have generally similar clonal frequency distributions (Figs. 4 and 5), and thus similar diversity profiles (Fig. 3), deviations from this 'immunosignature of health' (Additional file 8) may signal technological differences between experimental workflows (i.e., library preparation, cell population, sequencing depth). This may possibly prevent comparative repertoire analyses between any samples (be it healthy or diseased/infected) obtained from different laboratories [58]. Diversity profiles may, therefore, become a valuable bioinformatical tool for technological benchmarking and normalization of immune repertoire sequencing in the future, enabling cross-laboratory comparisons and meta-analyses, which would be highly valuable in advancing the development of next-generation immunogenomics diagnostics.

We showed that diversity profiles were considerably robust to sequencing depth (*technological undersampling*; Additional files 6, 7, and 10). This is at least in part due to the frequency-dependence of the diversity measures used, which leads to a relatively fast saturation in case of the power law distribution of immune repertoires [9], thus minimizing the overestimation of diversity resulting from low-frequency clones, which could have arisen from PCR and sequencing error. For any diversity measure to be biologically meaningful, it is highly desirable that samples cover a large portion of the biological diversity. To correct for insufficient *biological sampling*, recent investigations have focused on good estimators of selected diversity measures [23, 80] or even the underlying clonal frequency distribution [81]. However, the statistical research for diversity estimators of large-scale immune repertoire data is still in its infancy and needs further development.

Similarity-based clustering of CDR3 or V(D)J sequences (also called clonotyping) represents a frequent pre-processing step in immune repertoire analyses [82]. Recently, it has been shown that diversity profiles can potentially be linked to such similarity clustering approaches [37]. While, the lack of somatic hypermutation in T cells renders T-cell clonotype definition straightforward, it is a non-resolved issue in the data analysis of B-cell repertoire data [82]. Further research, possibly driven by the application of phylogenetic analyses [83] to immune repertoire data, will be needed to determine a clonotype definition that best represents a sample’s functional immune receptor diversity [84] as well as antigen specificity [3].

## Conclusions

We have shown that high-throughput immune repertoire sequencing coupled to sequence-independent diversity profiling may enable the development of immunodiagnostic cross-patient comparisons of immune repertoires, thus advancing the fields of systems and personalized medicine [85]. In contrast to the use of *distinct* (and very rare) disease-indicative public clonal sequences, diversity profiles reflect to a large extent the *entire* immune repertoire, which may reflect immunological statuses and their associated repertoire evolution and dynamics more sensitively. Indeed, we detected a large amount of immunological status-specific information in sequence-independent clonal repertoire distributions. Our approach is largely independent of the number of samples and therefore highly scalable for clinical use [86]. Finally, since immune repertoires represent fingerprints of the current status of an immune response, our approach can also be applied as a diagnostic metric to diseased or infected individuals where the disease or infection-causing antigen is unknown [87].

## Additional files

Additional file 1:
**Sequencing read statistics of analyzed datasets.** Of note, for dataset 4, the number of CDR3s is slightly higher than the actual cell numbers (≈5000). It is most likely that the increased number of CDR3s was a result of PCR-introduced errors (due to the use of Taq rather than a high-fidelity polymerase). It is well established that raw sequencing data would drastically overestimate the number of unique clones and the only way to fully overcome this would be to apply sophisticated experimental and bioinformatic methods for error correction (see Shugay et al. [21] for more information). Such an advanced method was not available to any of the researchers (or ourselves) who generated the datasets used in this manuscript. Therefore, we decided to use the simple approach of singleton exclusion (CDR3s with abundance of 1 were excluded). Other publications, including our own, have also used replicates to determine much more strict cutoffs [9, 68, 79]. Of note, if cutting at CDR3 abundance equaling 5 (as others have done [68]), the number of unique CDR3s is 1538 (data not shown), thus being well below the number of 5000 sorted cells. However, the important thing to acknowledge is that the clonal frequency distributions of naïve B cells on the one hand and that of ASCs and PCs on the other hand are markedly different and in line with biological expectations of B-cell populations (Additional file 2). It is these differences that cause the compartments to cluster apart (Additional file 9). Additional file 2:
**Diversity and Evenness profiles of frequency distributions depicted in Additional file** **4** **.** Numerous profiles intersect in in silico and experimental datasets. **a** Diversity and Evenness profiles of distributions in Additional file 4a. Per parameter combination, 200 Zipf-distributions were simulated and are shown as boxplots; the variance of Diversity and Evenness profiles for any given parameter combination is low. **b** Diversity and Evenness profiles of clonal frequency distributions of Additional file 4b (dataset 1) are graphed by immunological status. **c** Diversity and Evenness profiles of clonal frequency distributions of Additional file 4c (dataset 2) are graphed by immunological status. **d** Diversity and Evenness profiles of clonal frequency distributions of Additional file 4d (dataset 3). **e** Diversity and Evenness profiles of clonal frequency distributions of Additional file 4e (dataset 4). *ASC* antibody-secreting cells, *NFBC* naïve follicular B cells, *PC* plasma cells. Additional file 3:
**Flowchart of the P-SVM algorithm.** Support vector machine analysis was performed using the potential support vector machine (P-SVM) [57], which combines linear classification (classification of immunological status) of Diversity and Evenness profiles with the selection of a minimal subset of alpha values achieving the highest prediction accuracy (feature selection). The goal criterion of classification performance was balanced prediction accuracy (BACC = (Sensitivity + Specificity)/2)). The classification performance was measured using nested leave-one-out cross-validation, where feature selection and hyperparameter selection were performed in the inner cross-validation loop independently of the test sample of the outer cross-validation loop. The inner loop was used to determine the combination of parameters that give the best classification performance: the cost parameter c was varied from 1 to 17 in five equally spaced steps and the regularization parameter ε was chosen as 2^i^ with i = −3, −2,…, 3, 4. In order to obtain compact models that only use a small set of features, all parameter combinations in the inner cross-validation loop for which more than three models exceeded an upper limit of 20 selected alpha values were rejected. *BACC* balanced prediction accuracy. Additional file 4:
**Simulated and experimental immune repertoire datasets are Zipf-like distributed as evidenced by the near-linear relation between the logarithm of the clonal (CDR3) frequency and logarithm of the clonal rank.** **a** Zipf-distributions were simulated using the Zipf-R package with the parameter combinations (Zipf-α: 0.01, 0.1, 0.9; Zipf-B: 0.0001, 0.001, 0.01, 0.1). For further details regarding simulations, please refer to *Methods*. **b** CDR3 clonal frequency distributions of dataset 1 [10]. Due to the size of dataset 1, only one, although representative, distribution per immunological status is shown. **c** CDR3 clonal frequency distributions of dataset 2 [7]. **d** CDR3 clonal frequency distributions of dataset 3 [16]. **e** Clonal frequency distributions of dataset 4 are shown. Of note, Zipf-like behavior increases with increasing sequence coverage (NFBCs). *ASC* antibody-secreting cell, *NFBC* naïve follicular B cell, *PC* plasma cell. Additional file 5:
**Proof that Diversity (** ^**α**^ ***D*** **) is divisible into species richness and Evenness (** ^**α**^ ***E*** **).**
Additional file 6:
**Diversity profiles are qualitatively and quantitatively robust to varying sampling depth. a** Assessment of qualitative robustness to technological undersampling: the probability (color-coded, ranging from 0 [no intersection for no simulation run] to 1 [always intersection for all simulation runs]) of the intersection of profiles of Zipf distributions simulated using varying Zipf-α [a] (0.01,0.1,0.5,0.9) and Zipf-B [b] (0.0001, 0.001,0.01, 0.1, 1) parameters (Additional file 2) was assessed for 200 simulation runs per parameter combination and sampling depth (10–100 % of original sampling depth, 100 % = 10^6^ reads). Within heatmaps, each tile represents one parameter combination of Zipf-α and Zipf-B. The Rényi-alpha for all profiles ranged from α = 0 to α = 10 in steps of 0.2. **b** Assessment of quantitative robustness to technological undersampling: the mean ratio of pairs of Diversity profiles $\left(\frac{\varSigma_{\alpha}\frac{{}^{\alpha }D^i}{{}^{\alpha }D^j}}{n_{\alpha }}\right)$, where n_α_ is the number of alphas used (range, 0–10; step size, 0.2) and ^*α*^ *D* ^*i*^ and ^*α*^ *D* ^*j*^ are any two pairs of Diversity profiles, was assessed. **c** The Pearson correlation between the heatmaps of the complete datasets (100 % of simulated reads) in (a) and (b) and the undersampled ones (10–90 %). If undersampling had no influence on profiles, the correlation between the complete dataset and the undersampled ones was r = 1. Additional file 7:
**Evenness profiles are qualitatively and quantitatively robust to undersampling, though to a lesser extent than Diversity profiles.** **a** Assessment of qualitative robustness: the probability (color-coded, ranging from zero [no intersection for no simulation run] to one [always intersecting for all simulation runs]) of intersection of profiles of Zipf distributions simulated using varying Zipf-α [a] (0.01, 0.1, 0.5, 0.9) and Zipf-B [b] (0.0001, 0.001, 0.01, 0.1,1) parameters (Additional file 2) was assessed for 200 simulation runs per parameter combination and sampling depth (10–100 % of original sampling depth, 100 % = 10^6^ reads). Within heatmaps, each tile represents one parameter combination of Zipf-α and Zipf-B. The Rényi-alpha for all profiles ranged from α = 0 to α = 10 in steps of 0.2. **b** Assessment of quantitative robustness: the pairwise ratio of pairs of Evenness profiles $\left(\frac{\varSigma_{\alpha}\frac{{}^{\alpha }E^i}{{}^{\alpha }E^j}}{n_{\alpha }}\right)$, where n_α_ is the number of alphas used (range, 0–10; step size, 0.2) and ^*α*^ *E* ^*i*^ and ^*α*^ *E* ^*j*^ are any two pairs of Evenness profiles, was assessed. **c** The Pearson correlation between the heatmaps of the complete datasets (100 % of simulated reads) in (a) and (b) and the undersampled ones (10–90 %). If undersampling had no influence on profiles, the correlation between the complete dataset and the undersampled ones should be r = 1. Additional file 8:
**Diversity and Evenness profiles of dataset 3 (BCR, baseline versus influenza vaccination) do not cluster by sampling time point but cluster apart from cluster profiles of dataset 2 (BCR, healthy versus CLL).** **a** Hierarchical clustering of Diversity profiles of dataset 2 (healthy versus CLL) and dataset 3 (baseline versus influenza vaccination) was performed based on Euclidean distance and visualized using heatmaps. The heatmap depicts the pairwise distance coefficients of all profiles determined (see *Methods* for further details). Row colors describe dataset origin (dataset 2, red; dataset 3, blue), whereas column colors describe the different immunological statuses contained in datasets 2 (healthy, blue; CLL, red) and 3 (baseline, green; day 7, brown; day 21, violet). **b** Analogous analyses to (a) using Evenness profiles and correlation distance were performed. Additional file 9:
**Naïve follicular B cells cluster apart from antibody secreting cells.** **a** Hierarchical clustering of dataset 4 Diversity profiles was performed based on Euclidean distance and visualized using dendrograms. The respective sorted B-cell populations are color-coded. For more information on dataset 4, please refer to *Methods*. Of note, we recently showed that the sequencing depth achieved by us is sufficient to accurately represent murine repertoire diversity [9]. **b** Hierarchical clustering of dataset 4 Evenness profiles was performed based on correlation-based distance and visualized using dendrograms. The respective sorted B-cell populations are color-coded. Additional file 10:
**Hierarchical clustering of Diversity profiles is more robust to varying sampling depth than that of Evenness profiles.** **a** Dendrograms of the hierarchical clustering of mean Diversity profiles from undersampled data (10–100 % of total data, 10^6^ reads) are drawn per each sampling stage. The same parameter combinations as in Additional file 4 were used (200 Zipf-distributions per sampling stage and parameter combination). Hierarchical clustering on Diversity profiles was performed using the Euclidean distance. The first three clusters as determined by similarity are color-coded. **b** Dendrograms of hierarchical clustering of mean Evenness profiles from undersampled data (10–100 % of total data, 10^6^ reads) are drawn per each sampling stage. The same parameter combinations as in Additional file 4 were used (200 Zipf distributions per sampling stage and parameter combination). Hierarchical clustering on Evenness profiles was performed using the Pearson correlation-based distance. The first three clusters as determined by similarity are color-coded. **c** Boxplots of the cophenetic correlations for each of the 200 Zipf distribution for each parameter combination per sampling state (10–90 % of total data, 10^6^ reads) and the tree from the complete data set (100 %) was determined to quantify the clustering robustness. The cophenetic correlation between trees from Diversity profiles exceeds r = 0.8 for both Diversity and Evenness profiles at 30 % sampling of the complete dataset. Additional file 11:
**Diversity (** ^**α=1**^ ***D*** **) and Evenness (** ^**α=1**^ ***E*** **)** ***Shannon values*** **classify TCR (Dataset 1) and BCR (Dataset 2) immune repertoires with lower prediction accuracy (BACC, >64 %) than the respective profiles (Table** **1** **).** BACCs were computed using nested leave-one-out cross-validation and were regarded as significant if *p* < 0.01. Legend: BACC (Sensitivity + Specificity)/2, balanced prediction accuracy. Please refer to *Materials and Methods* for more details. Additional file 12:
**Diversity (** ^**α=2**^ ***D*** **) and Evenness (** ^**α=2**^ ***E*** **)** ***Simpson-Index values*** **classify TCR (dataset 1) and BCR (dataset 2) immune repertoires with lower BACC (prediction accuracy, >64 %) than the respective profiles (Additional file** **11** **).** BACCs were computed using nested leave-one-out cross-validation and were regarded as significant if *p* < 0.01. *BACC* (Sensitivity + Specificity)/2, balanced prediction accuracy. Please refer to *Methods* for more details.

## Abbreviations

ASC: antibody-secreting cell
BACC: balanced accuracy (prediction accuracy)
BCR: B-cell receptor
bp: base pair
CGG: chicken gamma globulin
CLL: chronic lymphocytic leukemia
CDR3: complementarity determining region 3
FACS: fluorescence-activated cell sorting
HTS: high-throughput sequencing
NFBC: naïve follicular B cell
IMGT: ImMunoGeneTics
NP: 4-hydroxy-3-nitrophenylacetyl
PBMC: peripheral blood mononuclear cell
PC: plasma cell
PCR: polymerase chain reaction
P-SVM: Potential Support Vector Machine
SR: species richness
SHM: somatic hypermutation
SVM: support vector machine
TCR: T-cell receptor
Vβ: variable beta chain
VH: variable heavy chain
