# Supplementary material for: A bioinformatic framework for immune repertoire diversity profiling enables detection of immunological status
Source: Genome Med. 2015 May 28;7(1):49. doi: 10.1186/s13073-015-0169-8 (PMC4489130; doi:10.1186/s13073-015-0169-8)
Supplement: Additional file 1: — Sequencing read statistics of analyzed datasets. Of note, for dataset 4, the number of CDR3s is slightly higher than the actual cell numbers (≈5000). It is most likely that the increased number of CDR3s was a result of PCR-introduced errors (due to the use of Taq rather than a high-fidelity polymerase). It is well established that raw sequencing data would drastically overestimate the number of unique clones and the only way to fully overcome this would be to apply sophisticated experimental and bioinformatic methods for error correction (see Shugay et al. [21] for more information). Such an advanced method was not available to any of the researchers (or ourselves) who generated the datasets used in this manuscript. Therefore, we decided to use the simple approach of singleton exclusion (CDR3s with abundance of 1 were excluded). Other publications, including our own, have also used replicates to determine much more strict cutoffs [9, 68, 79]. Of note, if cutting at CDR3 abundance equaling 5 (as others have done [68]), the number of unique CDR3s is 1538 (data not shown), thus being well below the number of 5000 sorted cells. However, the important thing to acknowledge is that the clonal frequency distributions of naïve B cells on the one hand and that of ASCs and PCs on the other hand are markedly different and in line with biological expectations of B-cell populations (Additional file 2). It is these differences that cause the compartments to cluster apart (Additional file 9). [file 13073_2015_169_MOESM1_ESM.docx]

­­ Additional file 11

| **Dataset** | **Immunological phenotype** | **Average sample size (Total number of sequencing reads post pre-processing)** | **Average number of unique CDR3s** |
| --- | --- | --- | --- |
| Dataset 1 | Baseline-CD4  Baseline 2-CD4  Baseline 12-CD4  Healthy-CD8  Month 2-CD8  Month 12-CD8 | 7094511  2619309  6304596  11723210  6117957  5796318 | 141701.5  3143.913  66483.48  221204.1  12848.26  167611.1 |
| Dataset 2 | Healthy  CLL | 45325  27322.8 | 3809.2  361.6364 |
| Dataset 3 | Baseline  Day 7  Day 21 | 8087.5  8472  7735 | 2219.857  2267.643  2255.714 |
| Dataset 4 | NFBC  ASC-IgG  ASC-IgM  PC-IgG  PC-IgM | 119055  40854  165380  3380  315975 | 44982  938  5890  134  6526 |
