# Supplementary figures and images for: A bioinformatic framework for immune repertoire diversity profiling enables detection of immunological status
Source: Genome Med. 2015 May 28;7(1):49. doi: 10.1186/s13073-015-0169-8 (PMC4489130; doi:10.1186/s13073-015-0169-8)

A

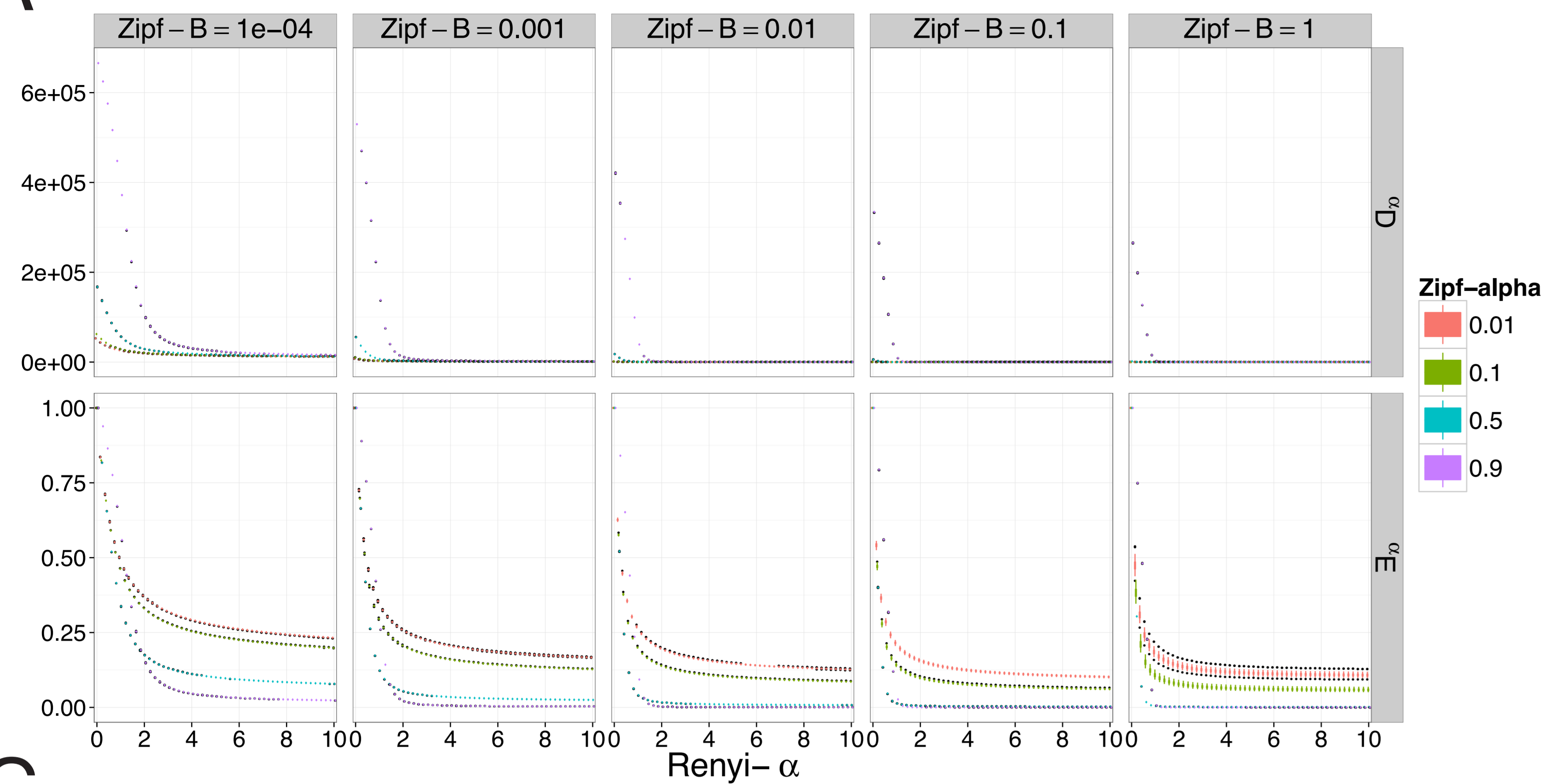

C

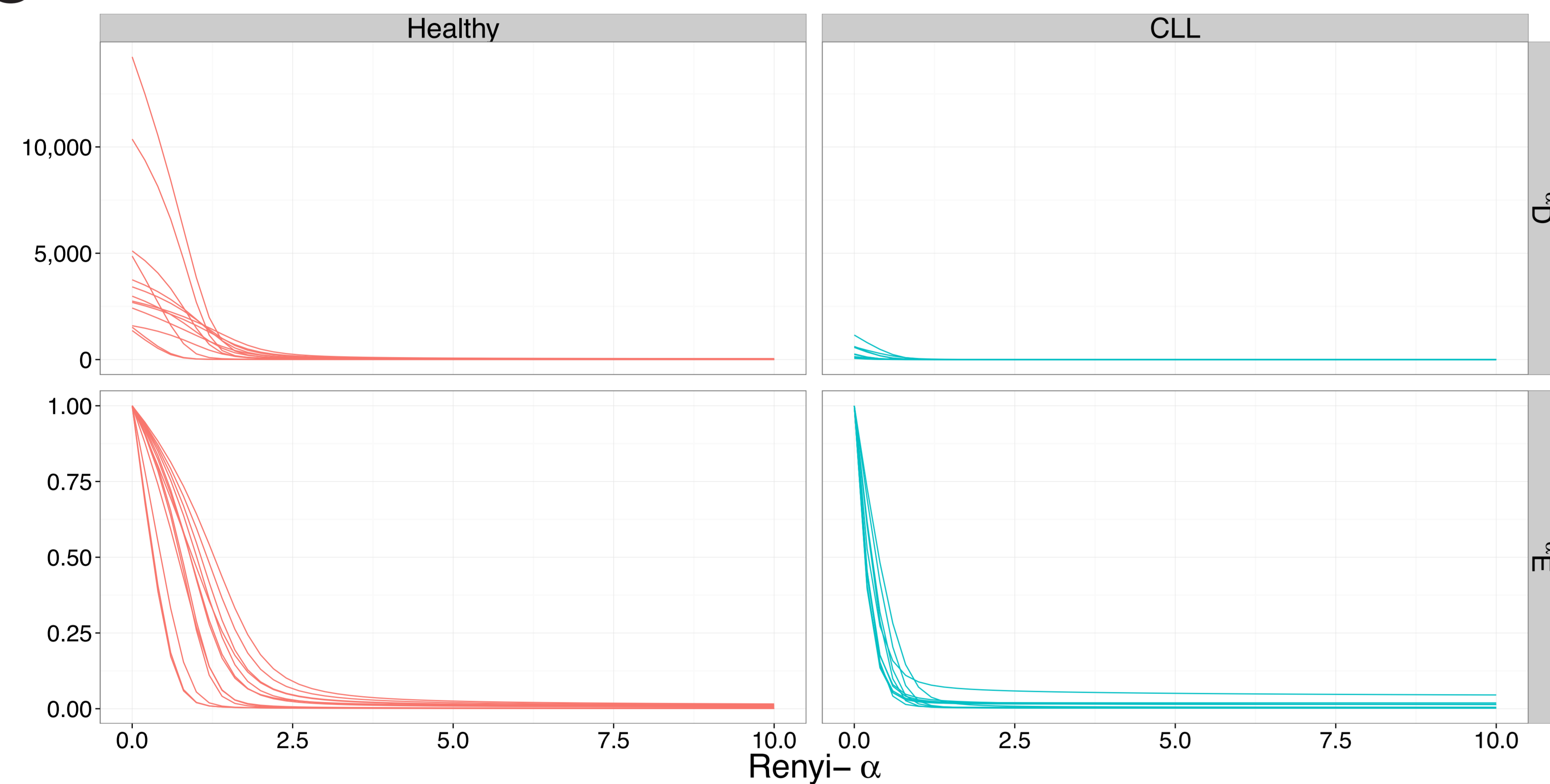

E

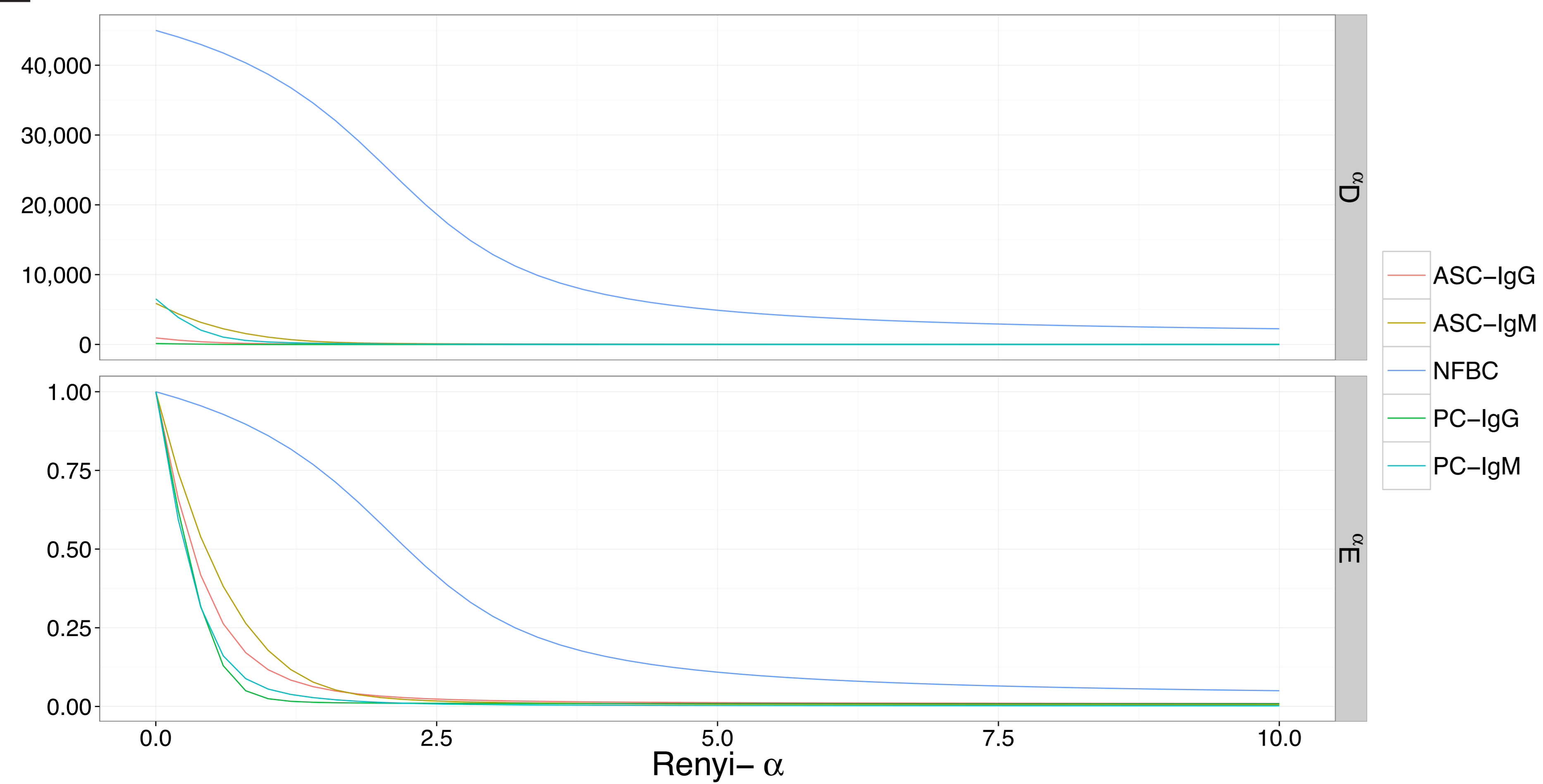

B

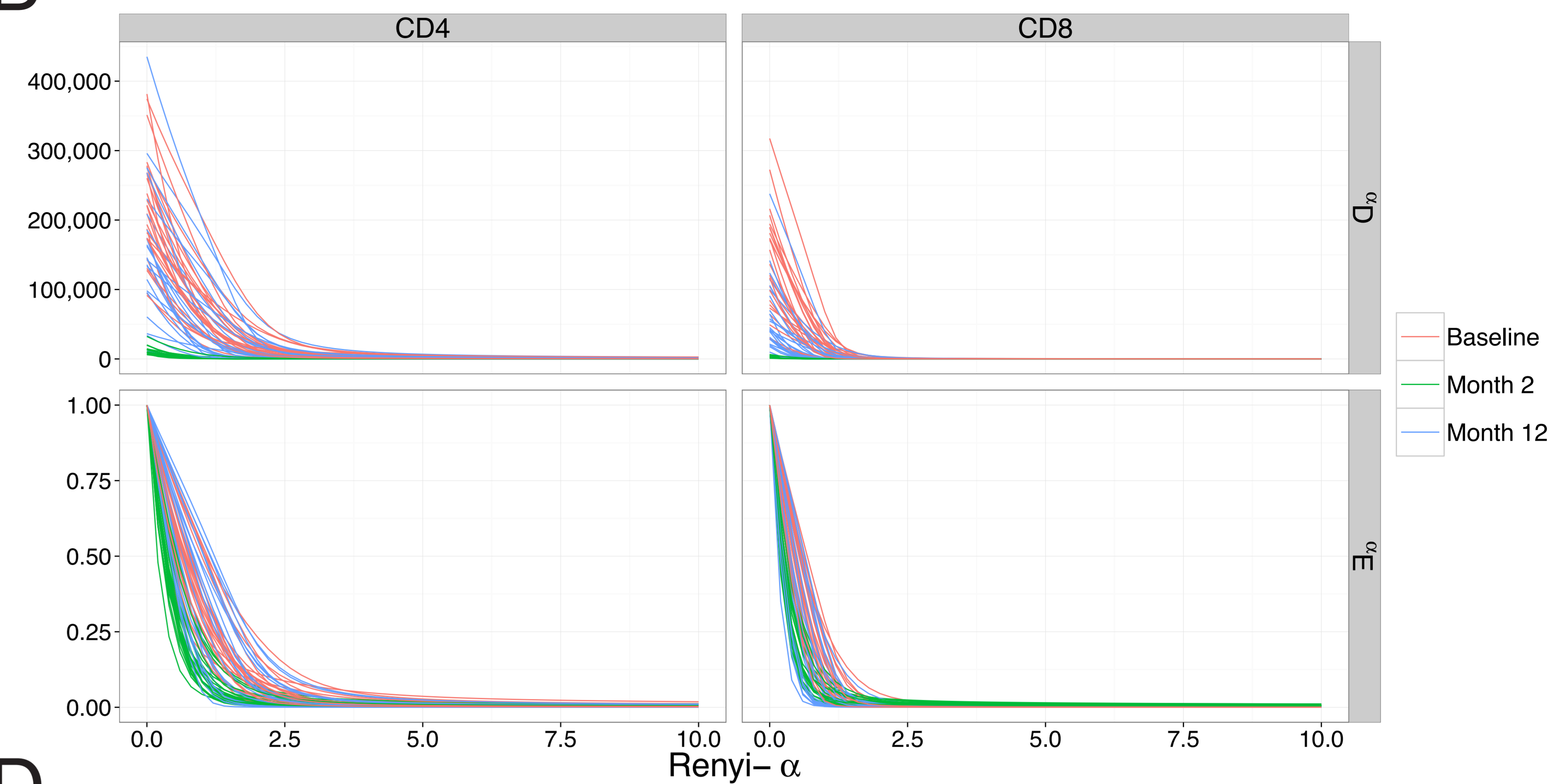

D

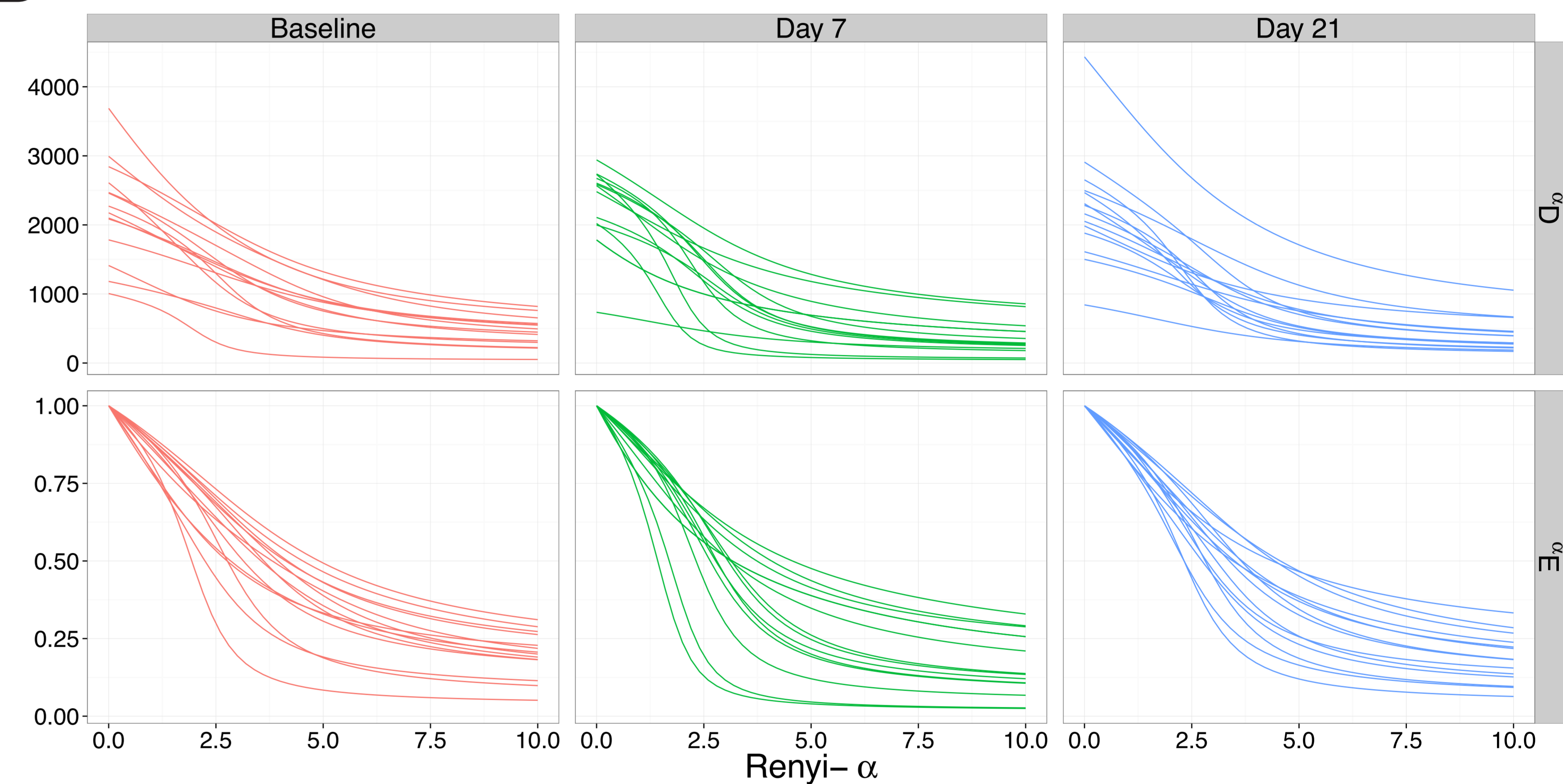

Supplement: Additional file 2: — Diversity and Evenness profiles of frequency distributions depicted in Additional file 4 . Numerous profiles intersect in in silico and experimental datasets. a Diversity and Evenness profiles of distributions in Additional file 4a. Per parameter combination, 200 Zipf-distributions were simulated and are shown as boxplots; the variance of Diversity and Evenness profiles for any given parameter combination is low. b Diversity and Evenness profiles of clonal frequency distributions of Additional file 4b (dataset 1) are graphed by immunological status. c Diversity and Evenness profiles of clonal frequency distributions of Additional file 4c (dataset 2) are graphed by immunological status. d Diversity and Evenness profiles of clonal frequency distributions of Additional file 4d (dataset 3). e Diversity and Evenness profiles of clonal frequency distributions of Additional file 4e (dataset 4). ASC antibody-secreting cells, NFBC naïve follicular B cells, PC plasma cells. [file 13073_2015_169_MOESM2_ESM.pdf]

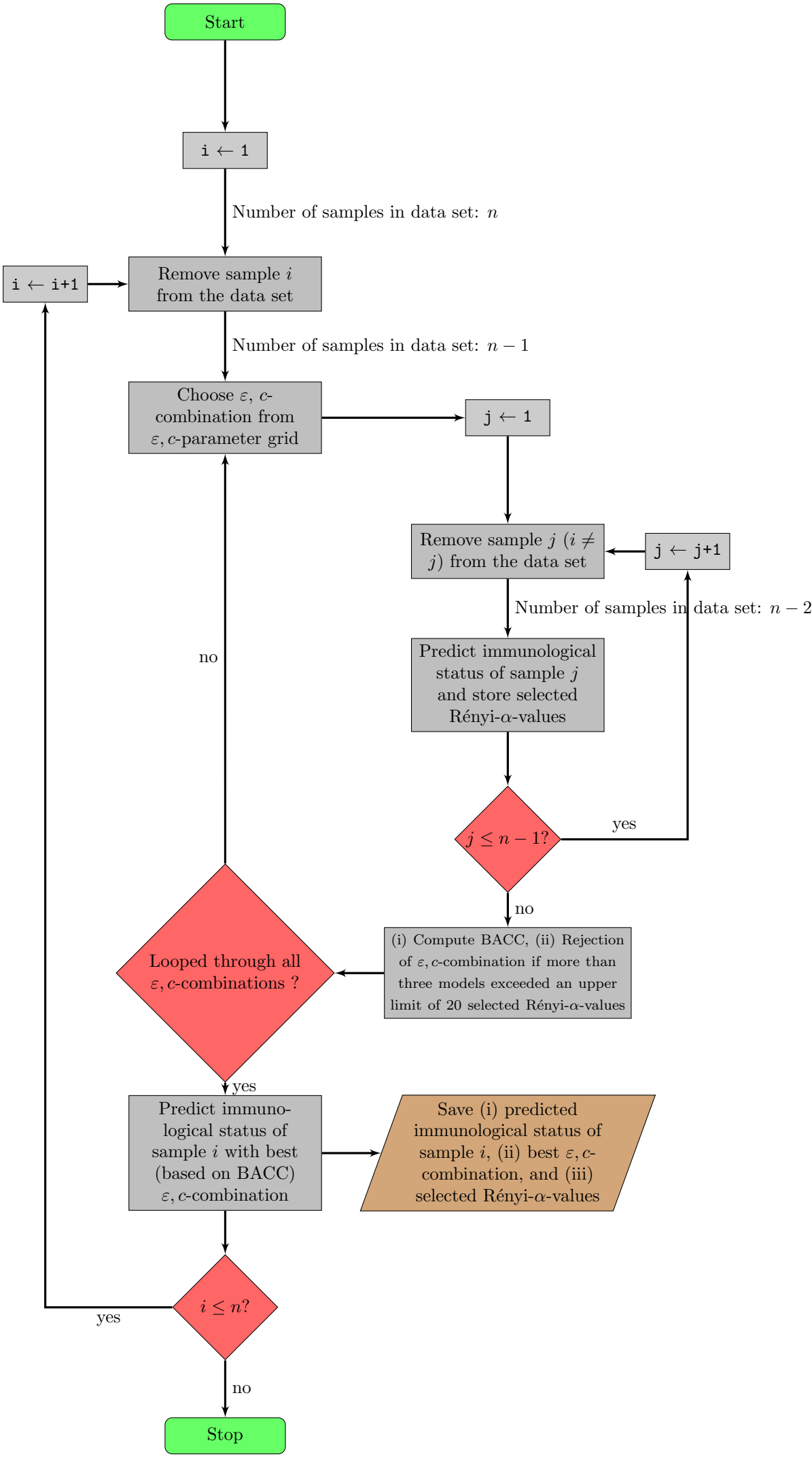

Supplement: Additional file 3: — Flowchart of the P-SVM algorithm. Support vector machine analysis was performed using the potential support vector machine (P-SVM) [57], which combines linear classification (classification of immunological status) of Diversity and Evenness profiles with the selection of a minimal subset of alpha values achieving the highest prediction accuracy (feature selection). The goal criterion of classification performance was balanced prediction accuracy (BACC = (Sensitivity + Specificity)/2)). The classification performance was measured using nested leave-one-out cross-validation, where feature selection and hyperparameter selection were performed in the inner cross-validation loop independently of the test sample of the outer cross-validation loop. The inner loop was used to determine the combination of parameters that give the best classification performance: the cost parameter c was varied from 1 to 17 in five equally spaced steps and the regularization parameter ε was chosen as 2i with i = −3, −2,…, 3, 4. In order to obtain compact models that only use a small set of features, all parameter combinations in the inner cross-validation loop for which more than three models exceeded an upper limit of 20 selected alpha values were rejected. BACC balanced prediction accuracy. [file 13073_2015_169_MOESM3_ESM.pdf]

**A**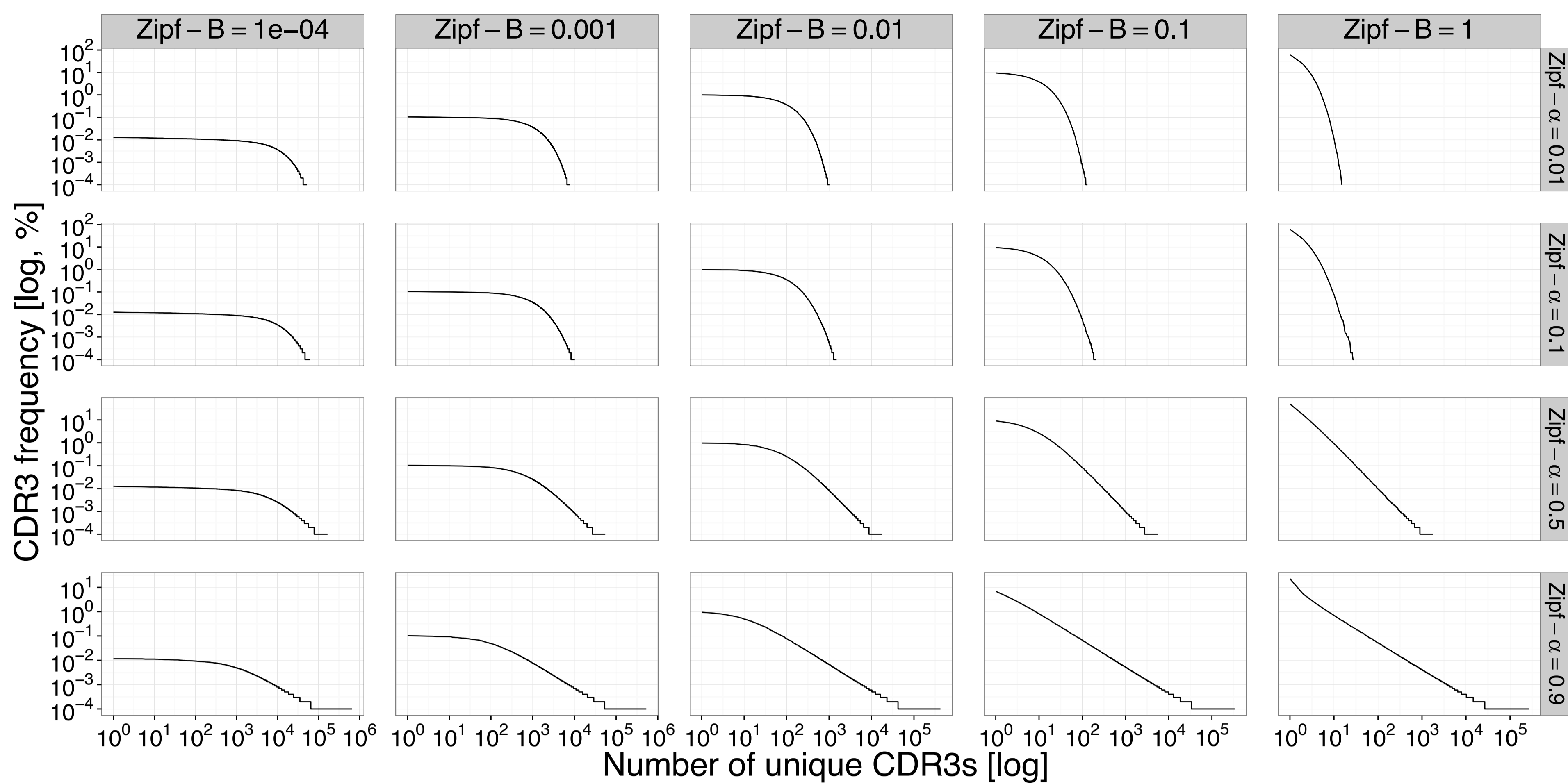**B**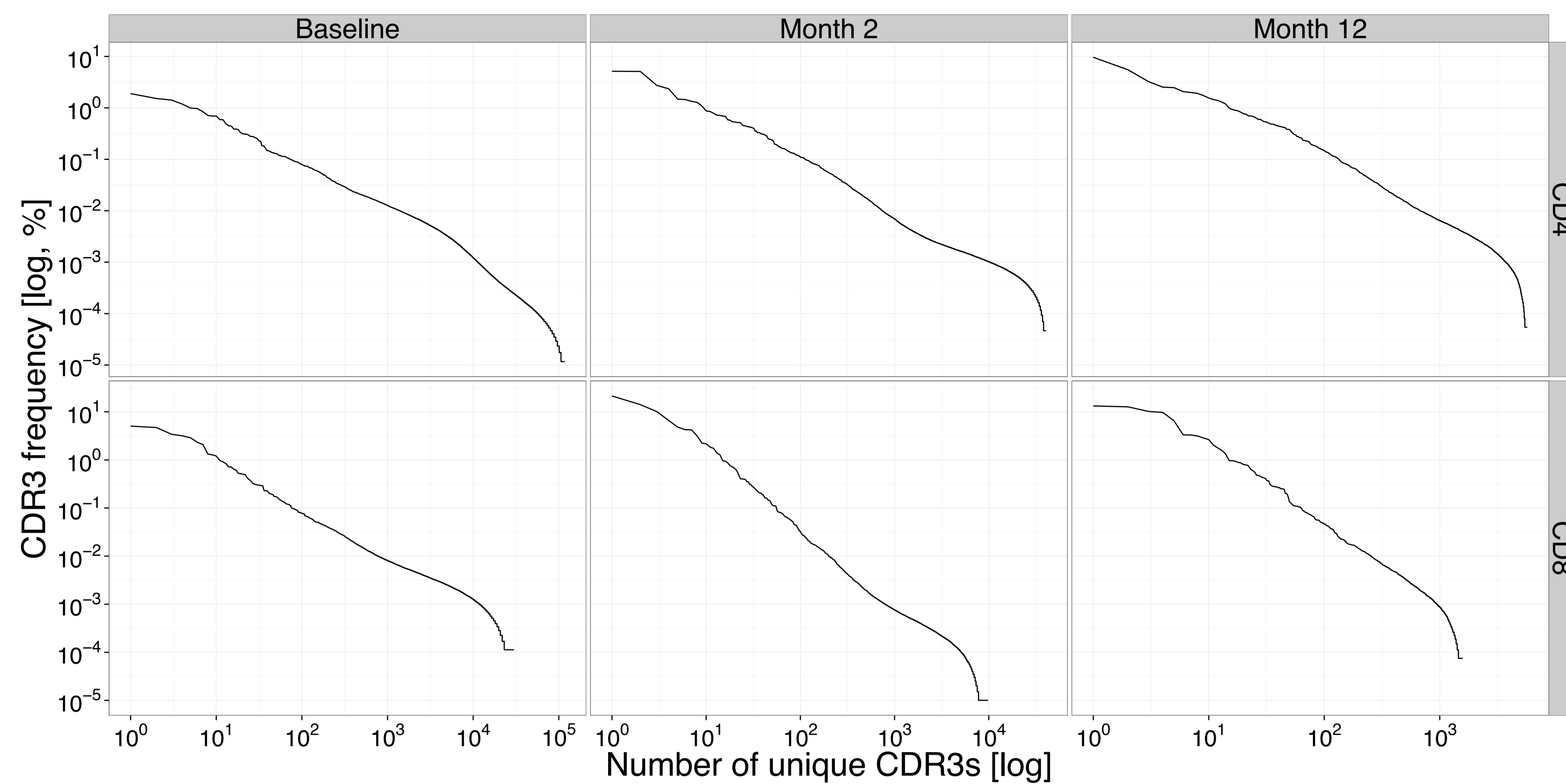**C**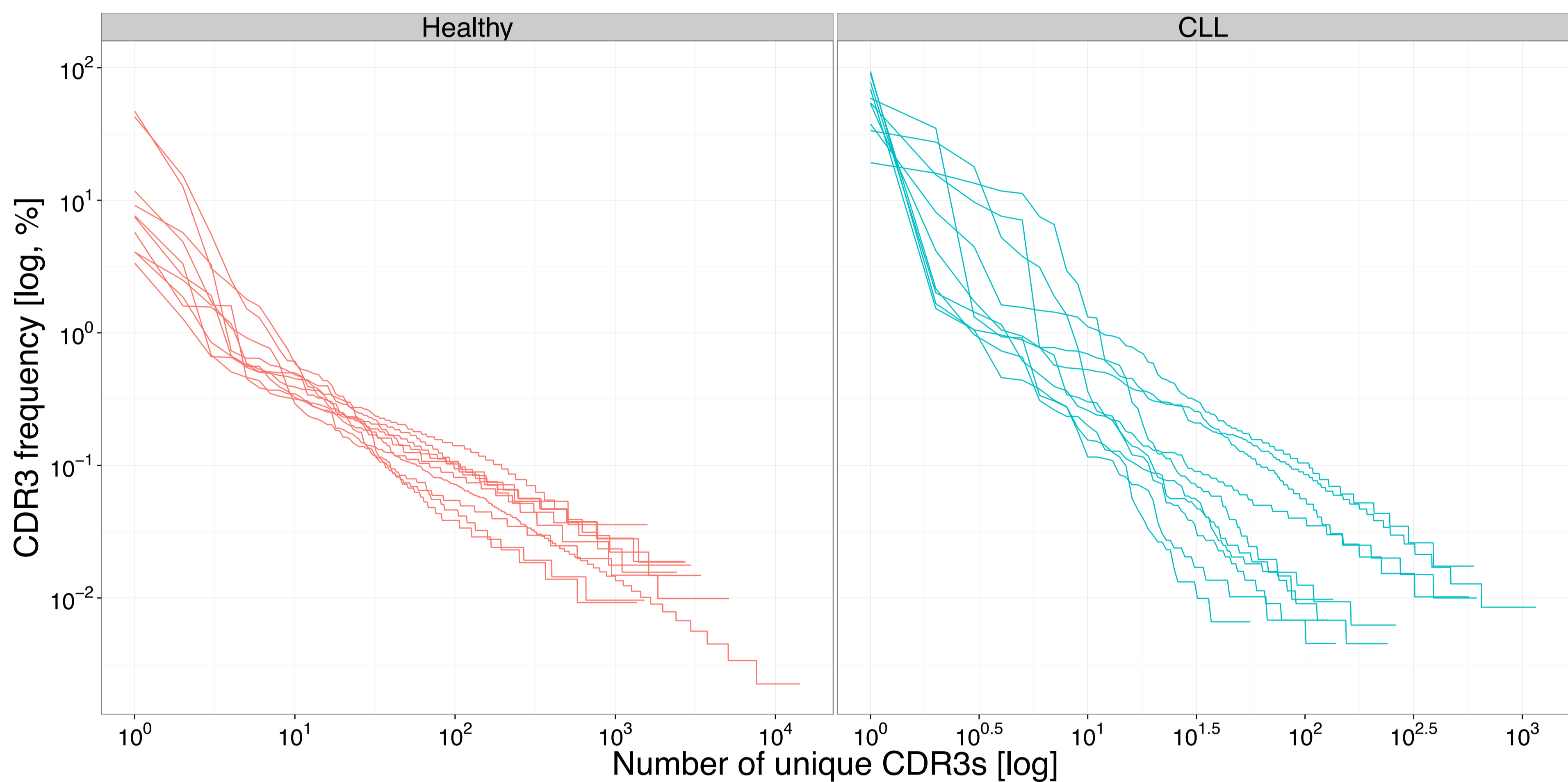**D**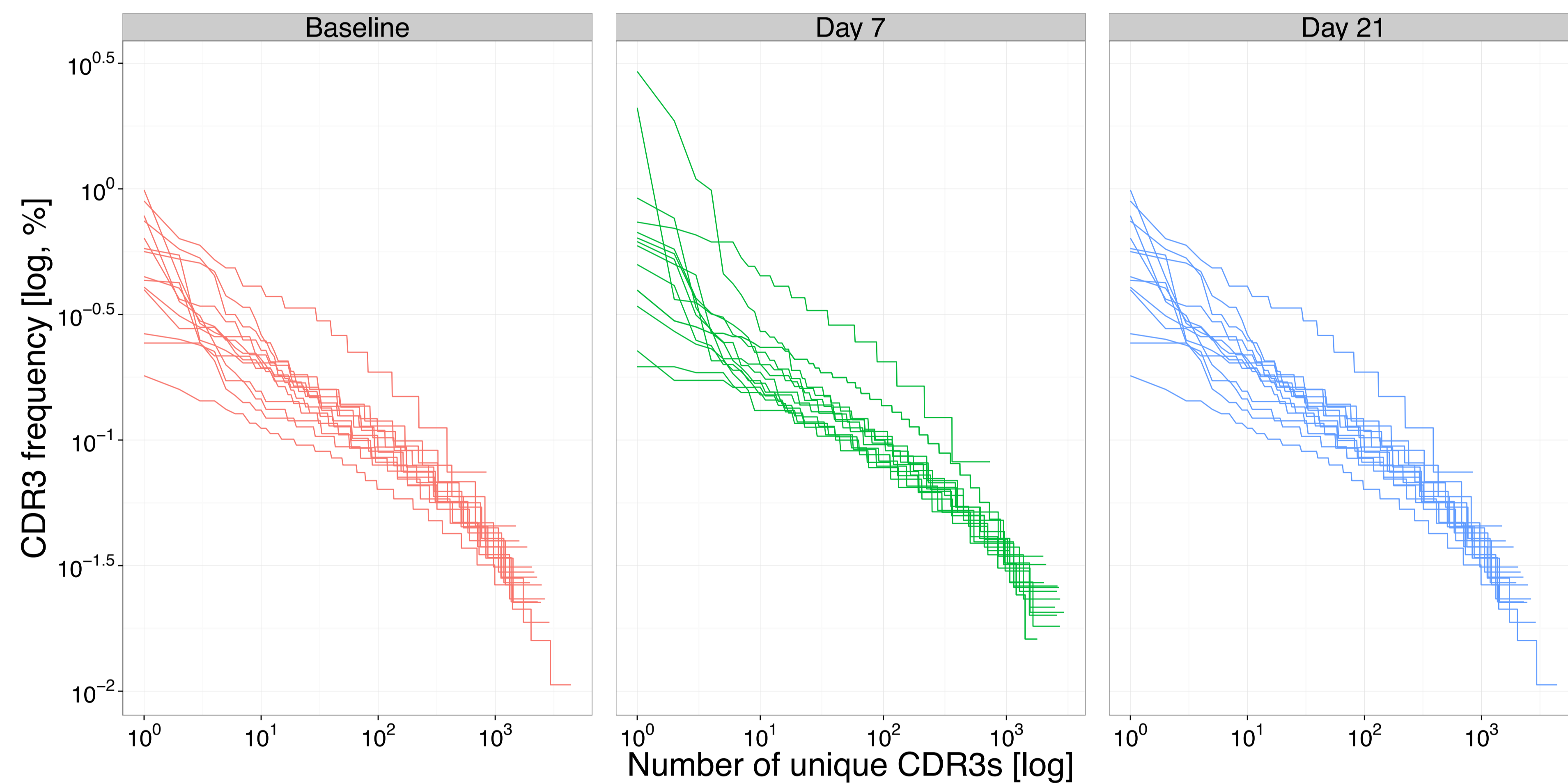**E**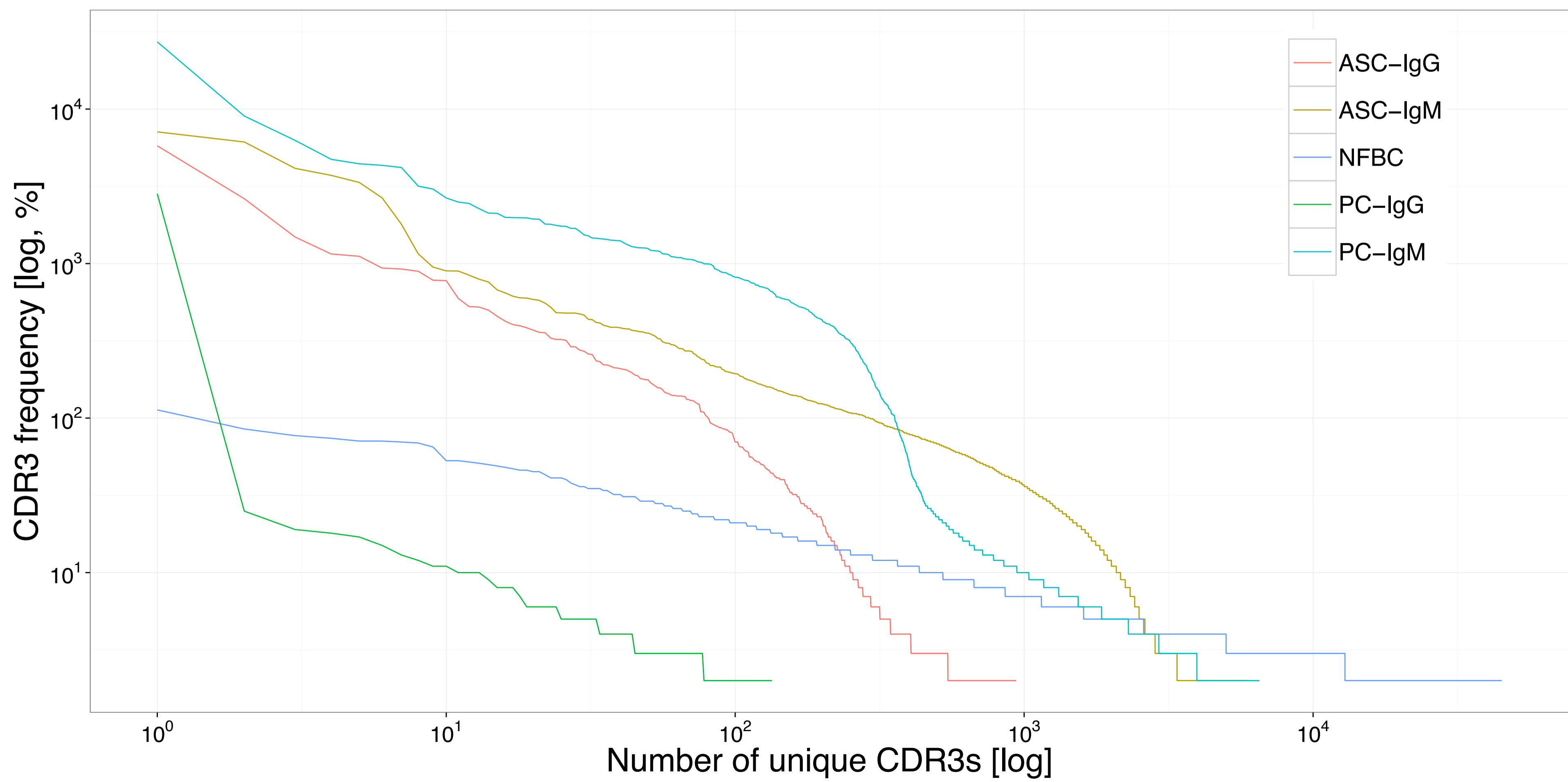

Supplement: Additional file 4: — Simulated and experimental immune repertoire datasets are Zipf-like distributed as evidenced by the near-linear relation between the logarithm of the clonal (CDR3) frequency and logarithm of the clonal rank. a Zipf-distributions were simulated using the Zipf-R package with the parameter combinations (Zipf-α: 0.01, 0.1, 0.9; Zipf-B: 0.0001, 0.001, 0.01, 0.1). For further details regarding simulations, please refer to Methods. b CDR3 clonal frequency distributions of dataset 1 [10]. Due to the size of dataset 1, only one, although representative, distribution per immunological status is shown. c CDR3 clonal frequency distributions of dataset 2 [7]. d CDR3 clonal frequency distributions of dataset 3 [16]. e Clonal frequency distributions of dataset 4 are shown. Of note, Zipf-like behavior increases with increasing sequence coverage (NFBCs). ASC antibody-secreting cell, NFBC naïve follicular B cell, PC plasma cell. [file 13073_2015_169_MOESM4_ESM.pdf]

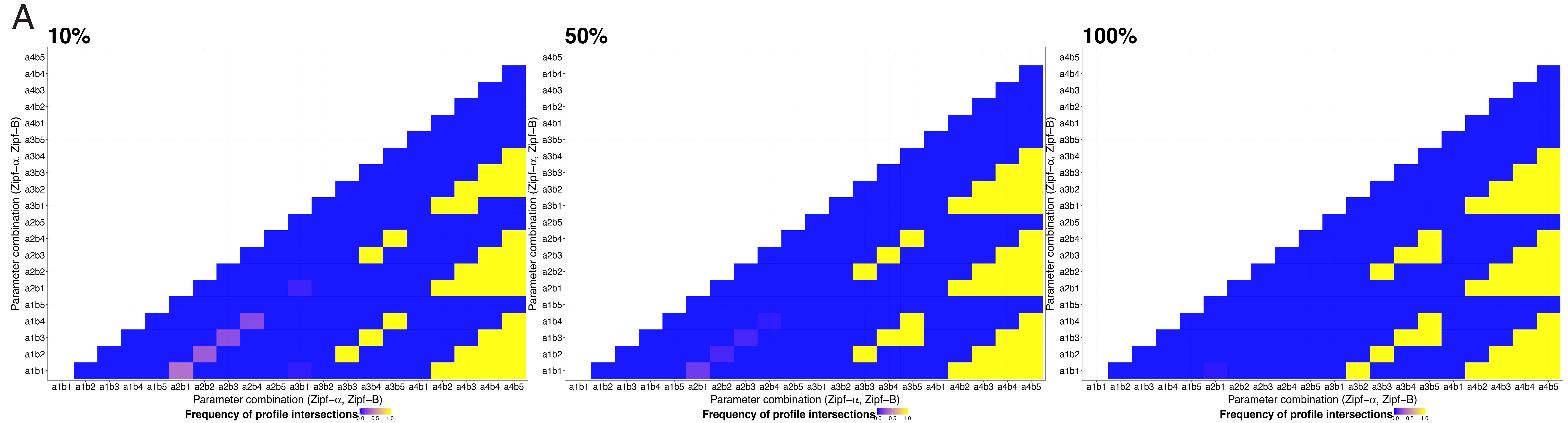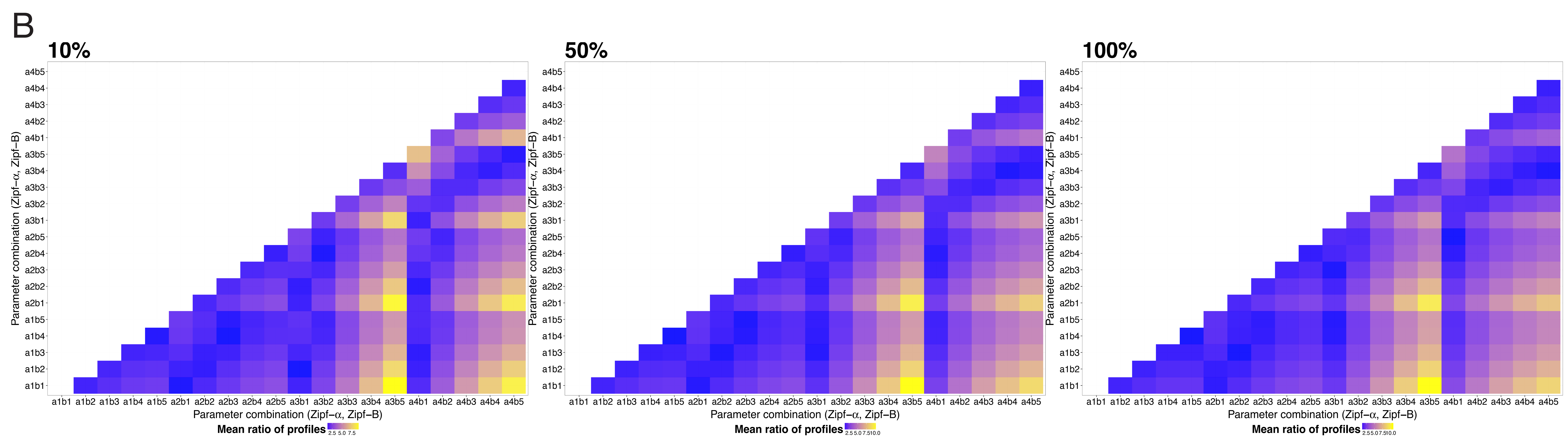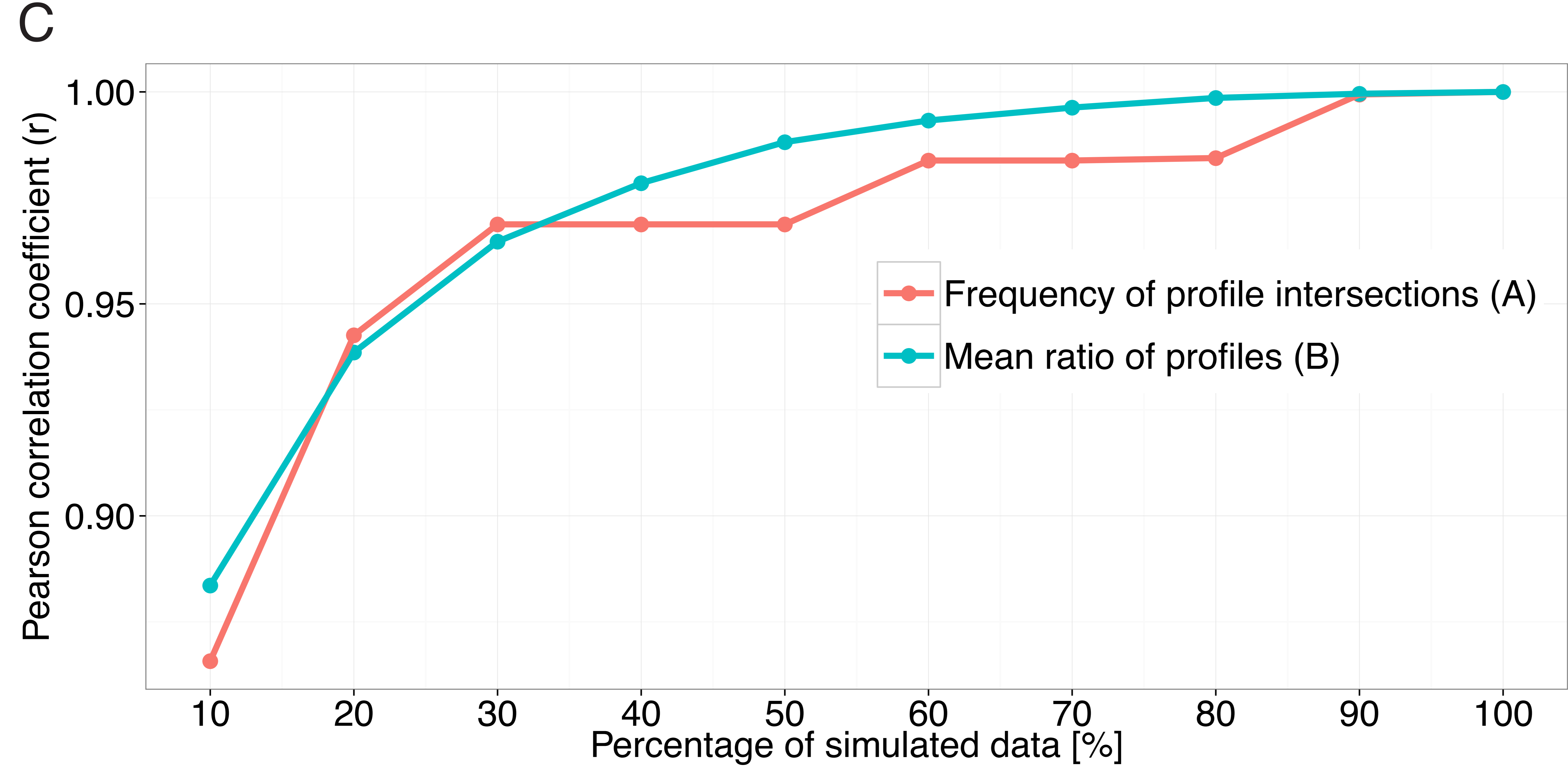

Supplement: Additional file 6: — Diversity profiles are qualitatively and quantitatively robust to varying sampling depth. a Assessment of qualitative robustness to technological undersampling: the probability (color-coded, ranging from 0 [no intersection for no simulation run] to 1 [always intersection for all simulation runs]) of the intersection of profiles of Zipf distributions simulated using varying Zipf-α [a] (0.01,0.1,0.5,0.9) and Zipf-B [b] (0.0001, 0.001,0.01, 0.1, 1) parameters (Additional file 2) was assessed for 200 simulation runs per parameter combination and sampling depth (10–100 % of original sampling depth, 100 % = 106 reads). Within heatmaps, each tile represents one parameter combination of Zipf-α and Zipf-B. The Rényi-alpha for all profiles ranged from α = 0 to α = 10 in steps of 0.2. b Assessment of quantitative robustness to technological undersampling: the mean ratio of pairs of Diversity profiles $\left(\frac{\varSigma_{\alpha}\frac{{}^{\alpha }D^i}{{}^{\alpha }D^j}}{n_{\alpha }}\right)$ΣαDiαDjαnα, where nα is the number of alphas used (range, 0–10; step size, 0.2) and α D i and α D j are any two pairs of Diversity profiles, was assessed. c The Pearson correlation between the heatmaps of the complete datasets (100 % of simulated reads) in (a) and (b) and the undersampled ones (10–90 %). If undersampling had no influence on profiles, the correlation between the complete dataset and the undersampled ones was r = 1. [file 13073_2015_169_MOESM6_ESM.pdf]

A

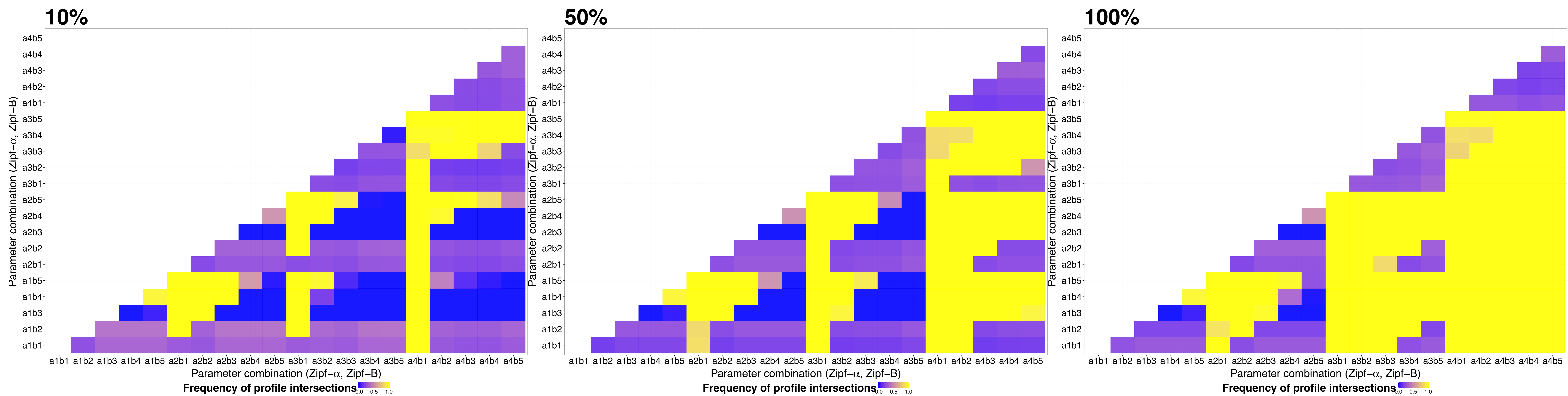

B

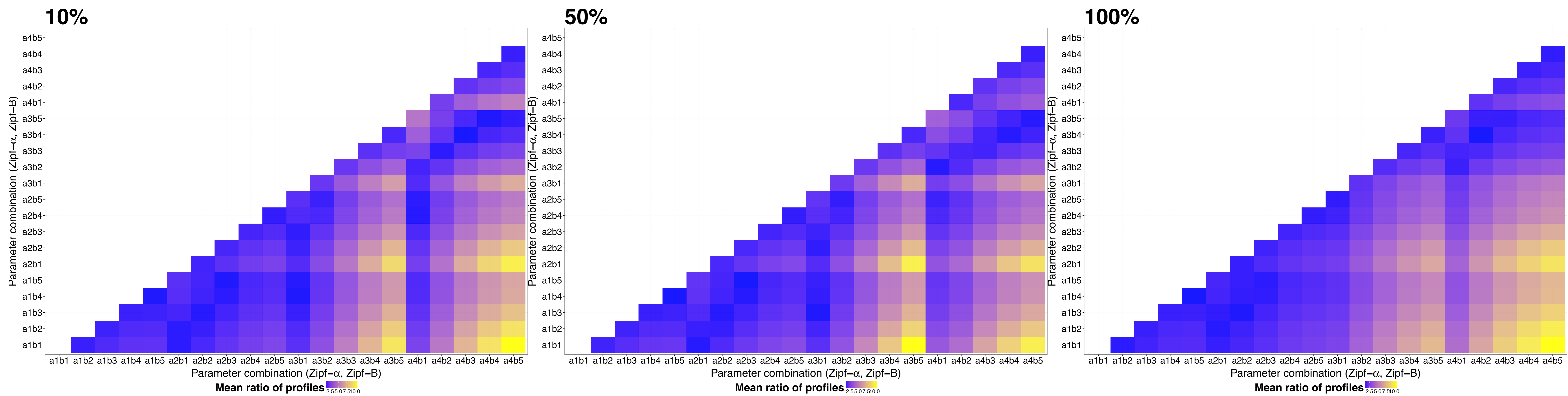

C

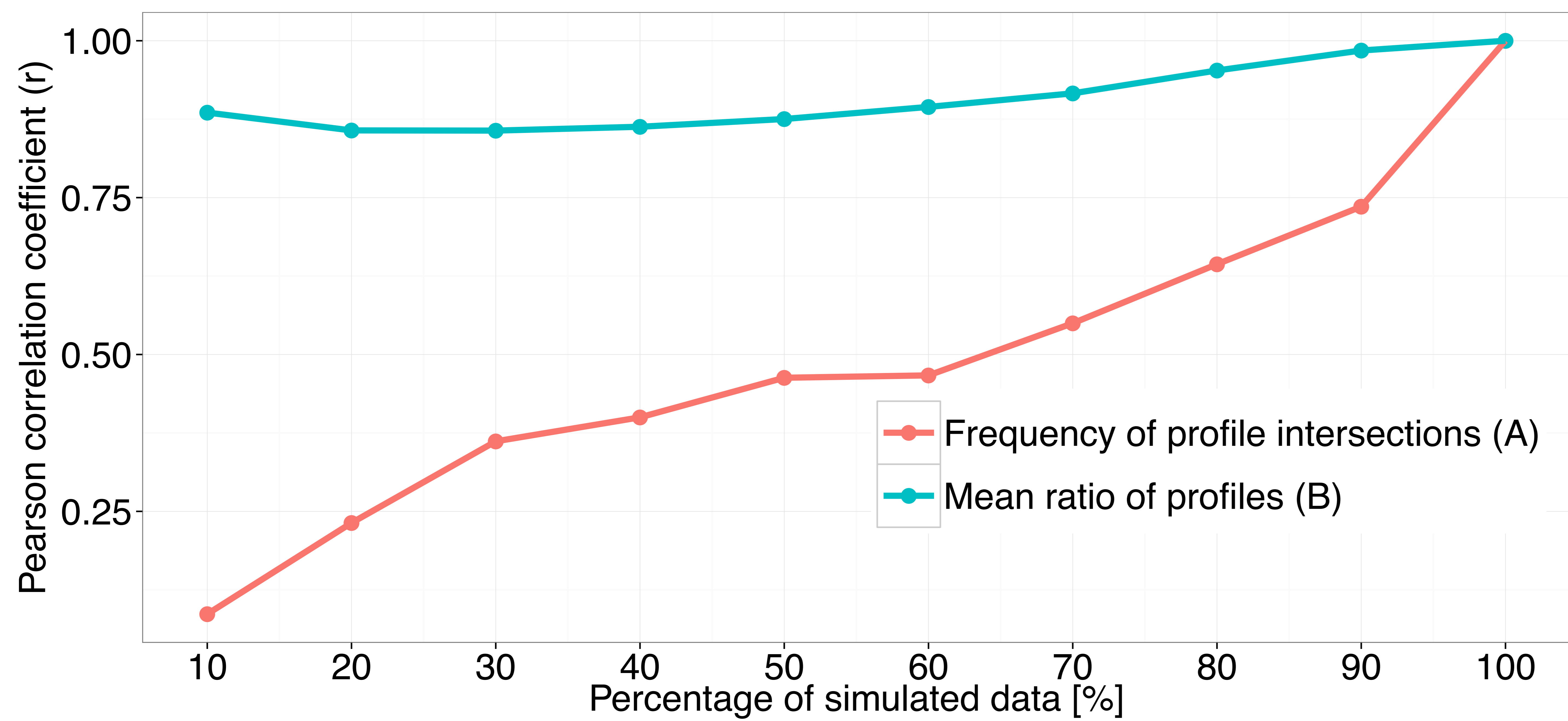

Supplement: Additional file 7: — Evenness profiles are qualitatively and quantitatively robust to undersampling, though to a lesser extent than Diversity profiles. a Assessment of qualitative robustness: the probability (color-coded, ranging from zero [no intersection for no simulation run] to one [always intersecting for all simulation runs]) of intersection of profiles of Zipf distributions simulated using varying Zipf-α [a] (0.01, 0.1, 0.5, 0.9) and Zipf-B [b] (0.0001, 0.001, 0.01, 0.1,1) parameters (Additional file 2) was assessed for 200 simulation runs per parameter combination and sampling depth (10–100 % of original sampling depth, 100 % = 106 reads). Within heatmaps, each tile represents one parameter combination of Zipf-α and Zipf-B. The Rényi-alpha for all profiles ranged from α = 0 to α = 10 in steps of 0.2. b Assessment of quantitative robustness: the pairwise ratio of pairs of Evenness profiles $\left(\frac{\varSigma_{\alpha}\frac{{}^{\alpha }E^i}{{}^{\alpha }E^j}}{n_{\alpha }}\right)$ΣαEiαEjαnα, where nα is the number of alphas used (range, 0–10; step size, 0.2) and α E i and α E j are any two pairs of Evenness profiles, was assessed. c The Pearson correlation between the heatmaps of the complete datasets (100 % of simulated reads) in (a) and (b) and the undersampled ones (10–90 %). If undersampling had no influence on profiles, the correlation between the complete dataset and the undersampled ones should be r = 1. [file 13073_2015_169_MOESM7_ESM.pdf]

A

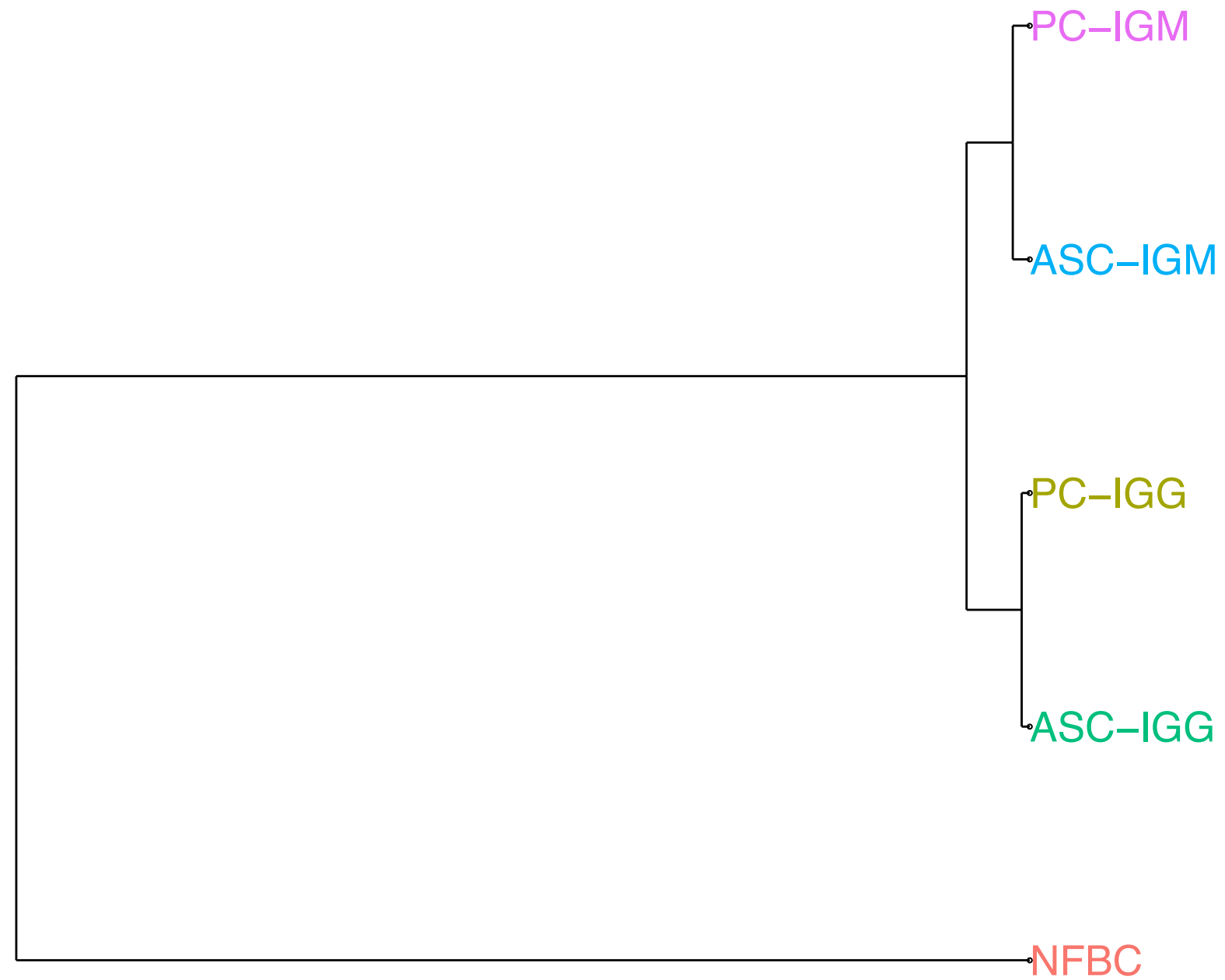

B

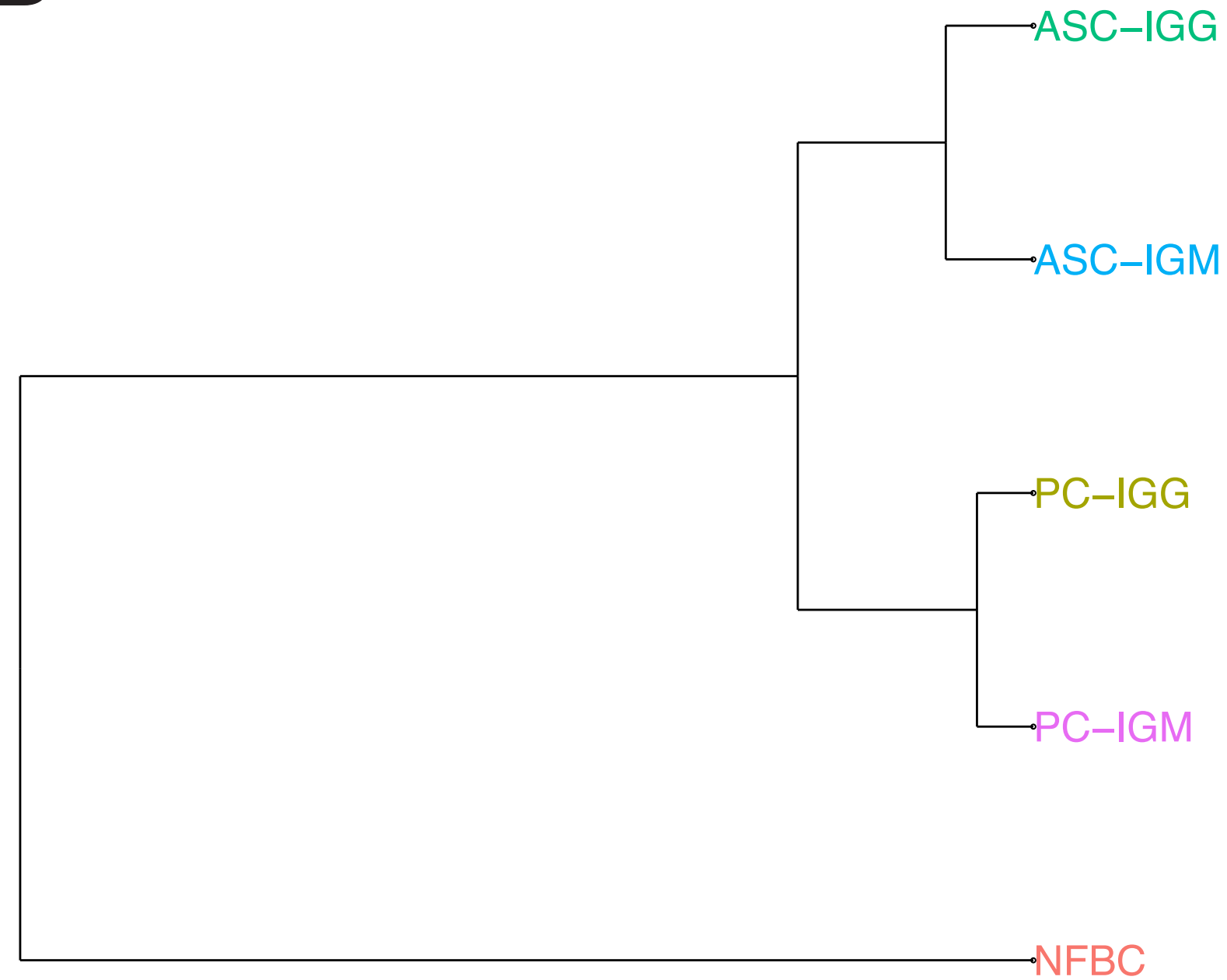

Supplement: Additional file 9: — Naïve follicular B cells cluster apart from antibody secreting cells. a Hierarchical clustering of dataset 4 Diversity profiles was performed based on Euclidean distance and visualized using dendrograms. The respective sorted B-cell populations are color-coded. For more information on dataset 4, please refer to Methods. Of note, we recently showed that the sequencing depth achieved by us is sufficient to accurately represent murine repertoire diversity [9]. b Hierarchical clustering of dataset 4 Evenness profiles was performed based on correlation-based distance and visualized using dendrograms. The respective sorted B-cell populations are color-coded. [file 13073_2015_169_MOESM9_ESM.pdf]

A

10%

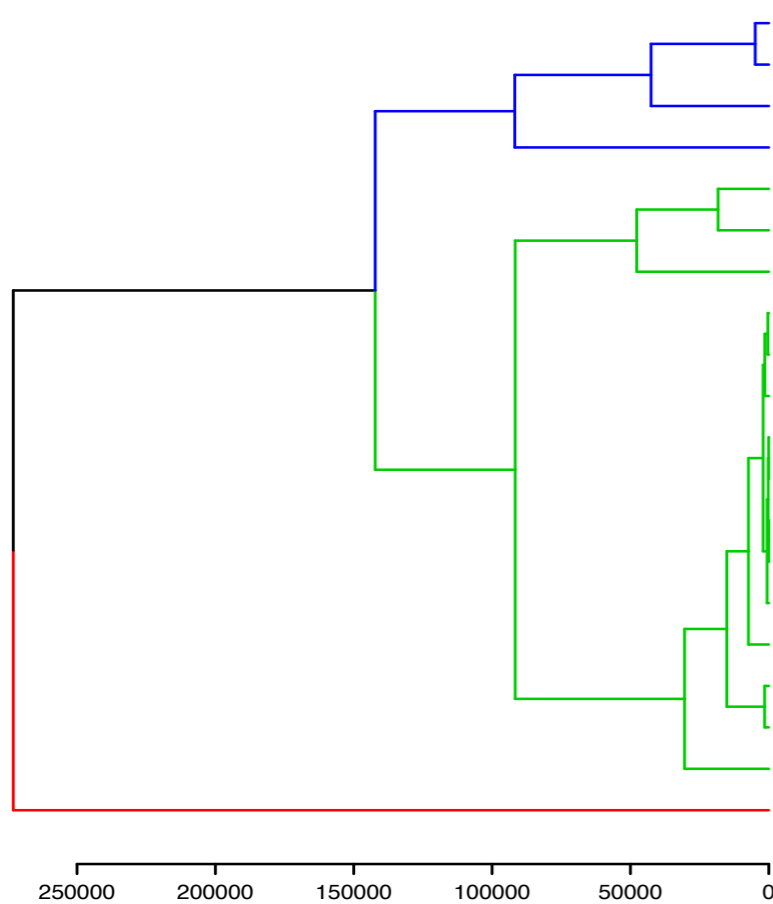

50%

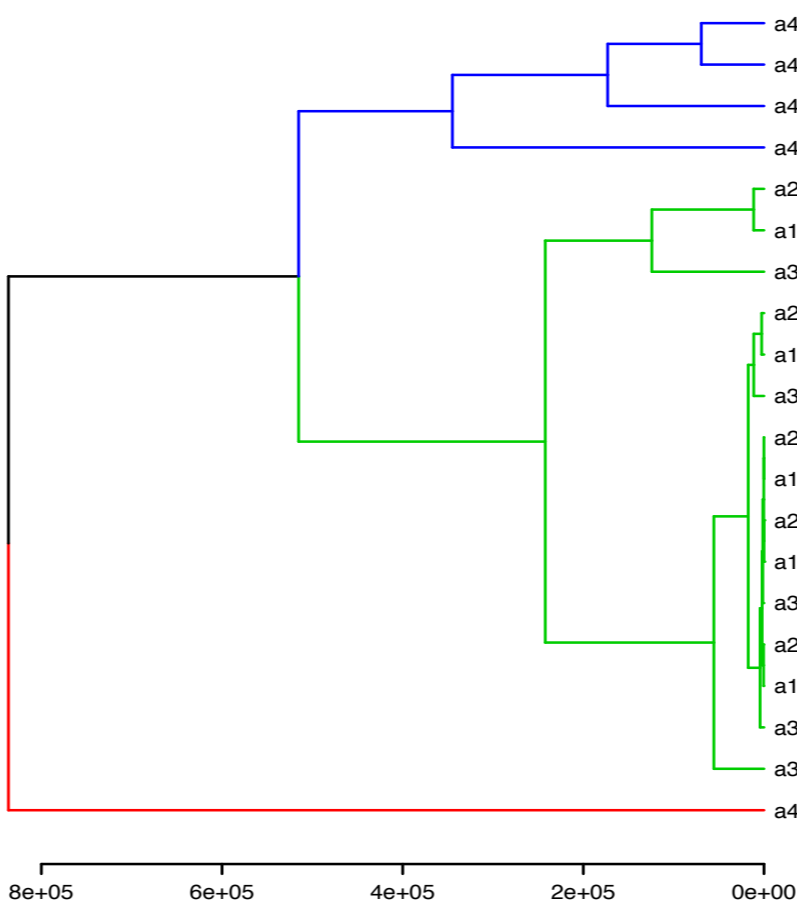

100%

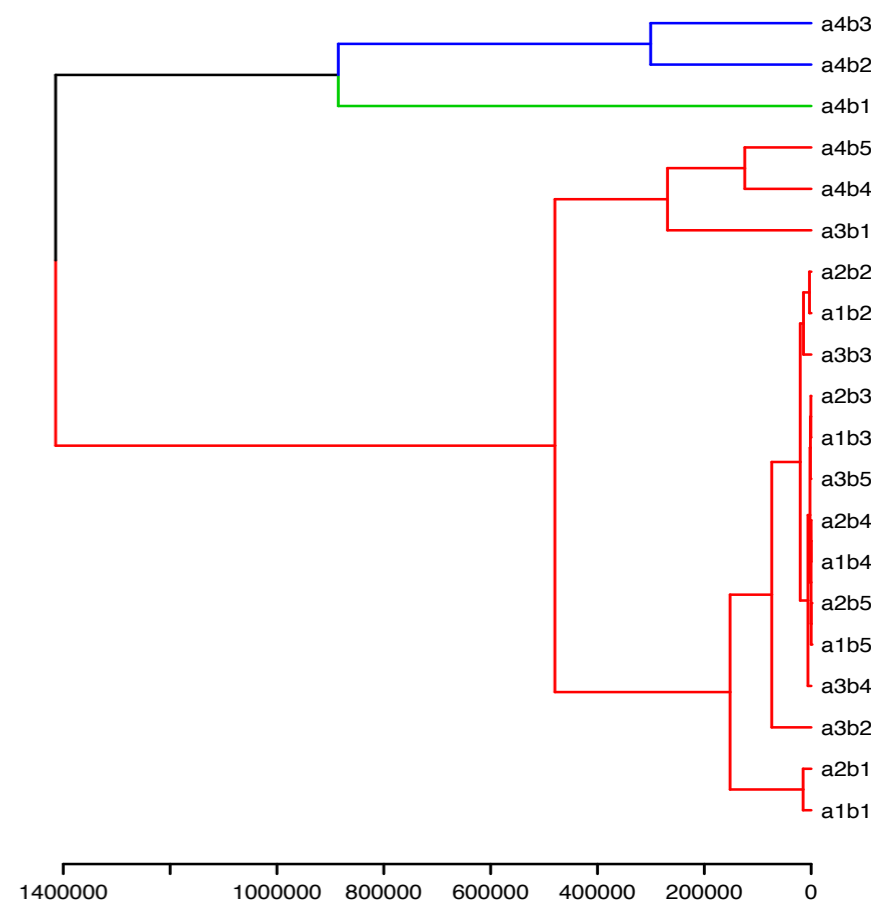

B

10%

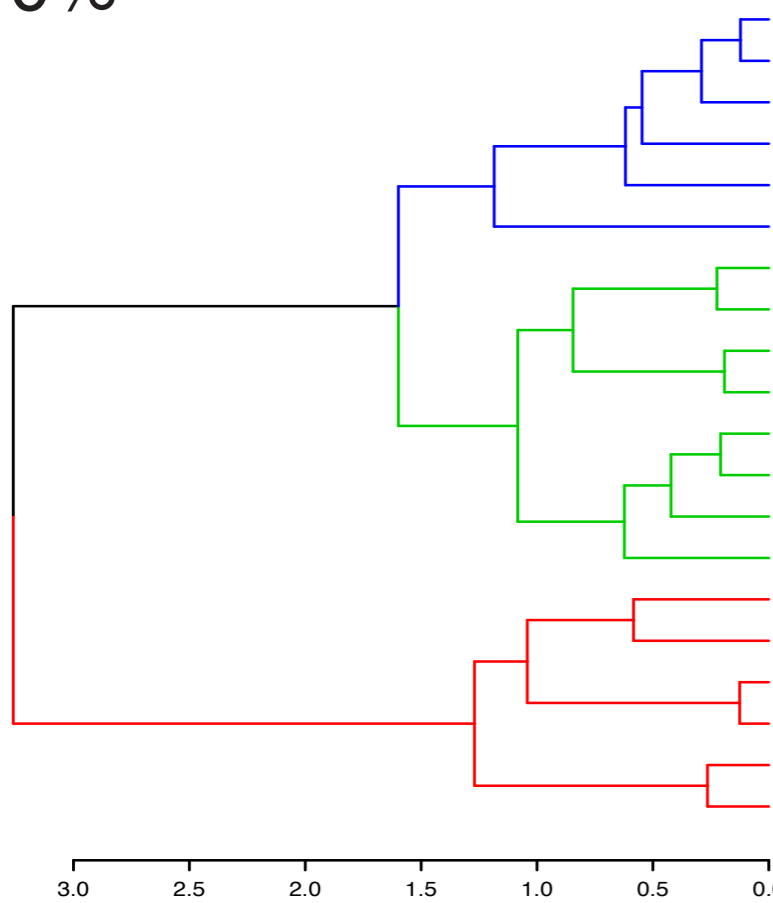

50%

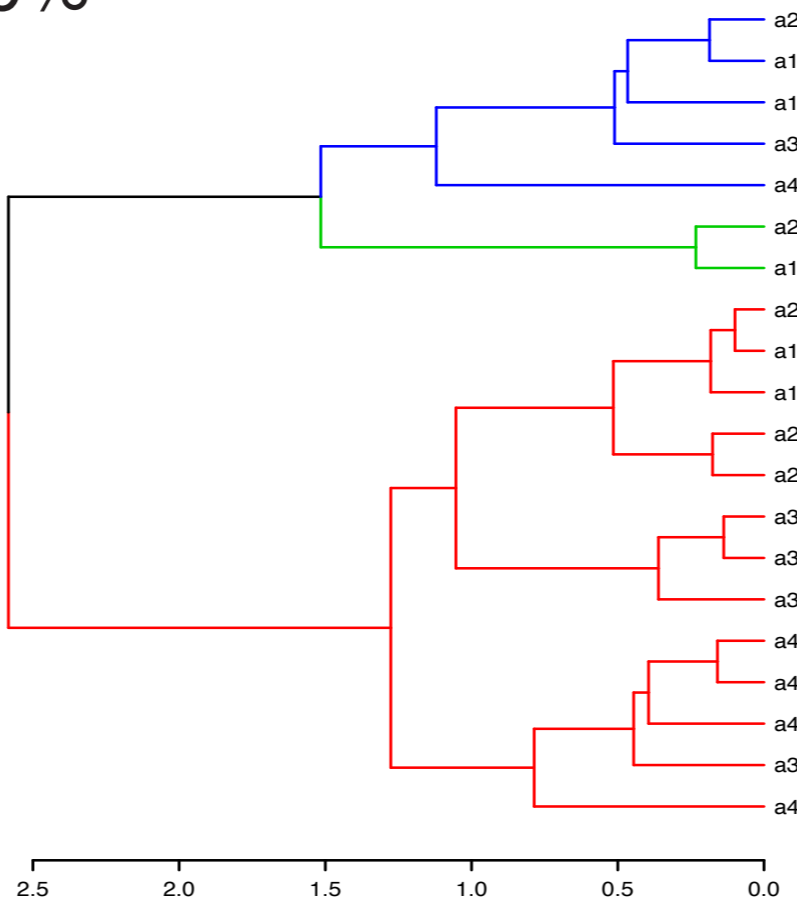

100%

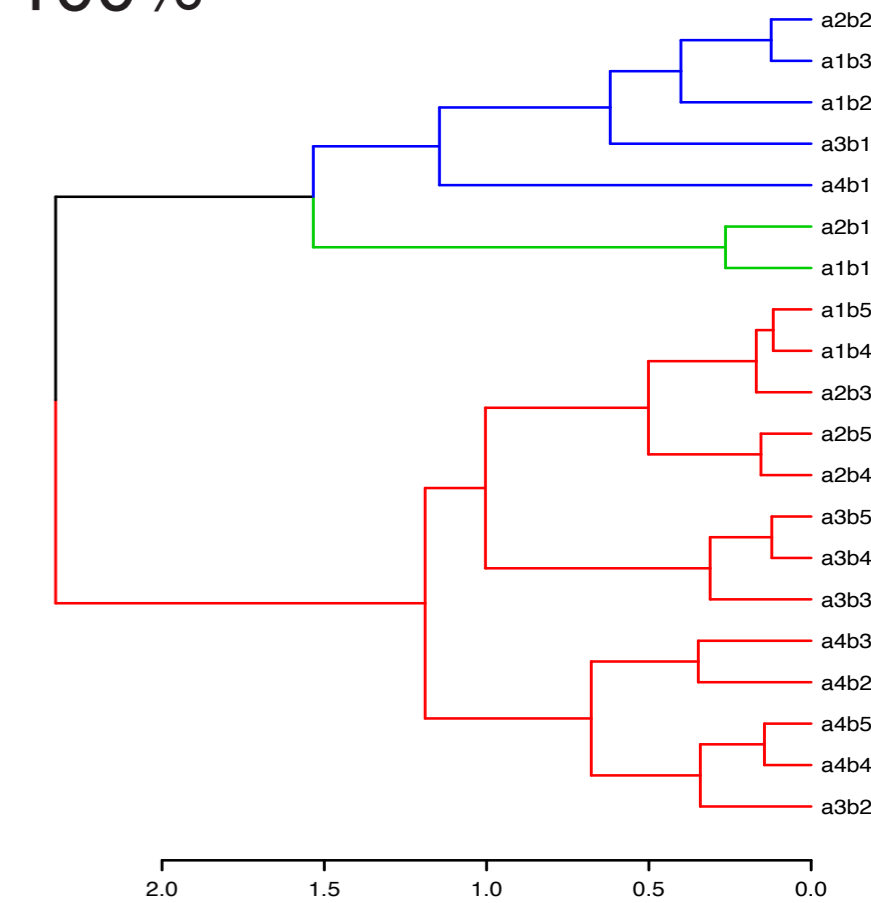

C

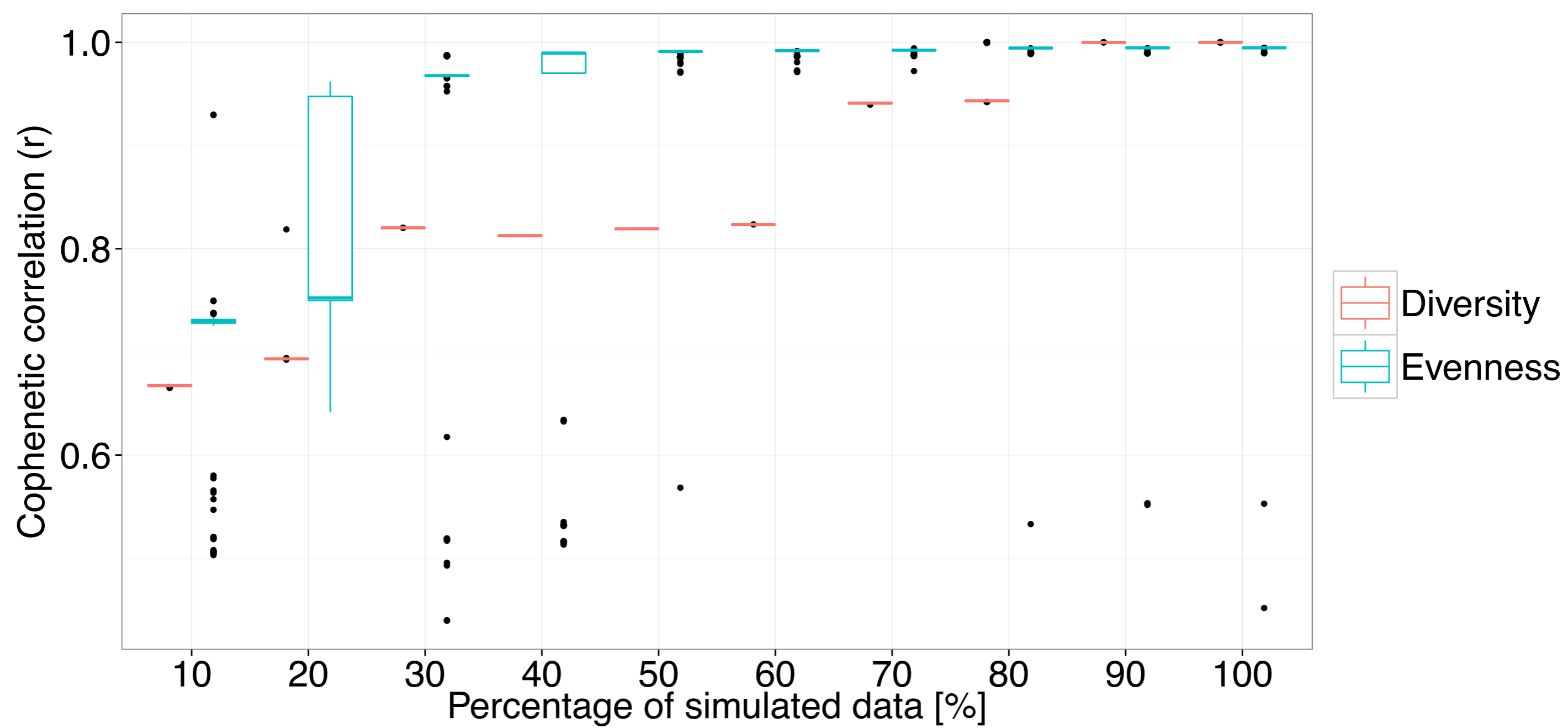

Supplement: Additional file 10: — Hierarchical clustering of Diversity profiles is more robust to varying sampling depth than that of Evenness profiles. a Dendrograms of the hierarchical clustering of mean Diversity profiles from undersampled data (10–100 % of total data, 106 reads) are drawn per each sampling stage. The same parameter combinations as in Additional file 4 were used (200 Zipf-distributions per sampling stage and parameter combination). Hierarchical clustering on Diversity profiles was performed using the Euclidean distance. The first three clusters as determined by similarity are color-coded. b Dendrograms of hierarchical clustering of mean Evenness profiles from undersampled data (10–100 % of total data, 106 reads) are drawn per each sampling stage. The same parameter combinations as in Additional file 4 were used (200 Zipf distributions per sampling stage and parameter combination). Hierarchical clustering on Evenness profiles was performed using the Pearson correlation-based distance. The first three clusters as determined by similarity are color-coded. c Boxplots of the cophenetic correlations for each of the 200 Zipf distribution for each parameter combination per sampling state (10–90 % of total data, 106 reads) and the tree from the complete data set (100 %) was determined to quantify the clustering robustness. The cophenetic correlation between trees from Diversity profiles exceeds r = 0.8 for both Diversity and Evenness profiles at 30 % sampling of the complete dataset. [file 13073_2015_169_MOESM10_ESM.pdf]
