# Supplementary material for: A bioinformatic framework for immune repertoire diversity profiling enables detection of immunological status
Source: Genome Med. 2015 May 28;7(1):49. doi: 10.1186/s13073-015-0169-8 (PMC4489130; doi:10.1186/s13073-015-0169-8)
Supplement: Additional file 5: — Proof that Diversity ( α D ) is divisible into species richness and Evenness ( α E ). [file 13073_2015_169_MOESM5_ESM.pdf]

**Additionale file 12**

*Diversity ($^{\alpha}D$) is divisable into Species Richness ($SR = {}^{\alpha=0}D$) and Evenness ($^{\alpha}E$).*

*Proof.* Let $f = \{f_i\}_{i=1,...,n}$ be the clonal frequency distribution of a given immune repertoire; $f_i$ is the clonal frequency of each clone and the species richness (number of unique clones) of the immune repertoire is $n$. Let $f_u$ be the uniform distribution of species richness $n$, wherein each clone is equally common ($f_{u,i} = \frac{1}{n}$ for all $i$ in $1, ..., n$). Taking advantage of the fact that the Rényi entropy ($^{\alpha}$RE) decomposes into the logarithm of the Species Richness (SR) and into the Rényi divergence ($^{\alpha}D(f||f_u)$), one gets:

$$^{\alpha}\mathrm{RE} = \frac{1}{1-\alpha}\log\left(\sum_{i=1}^{n} p_i^{\alpha}\right) \tag{1}$$

$$^{\alpha}\mathrm{RE} = \log(\mathrm{SR}) + {}^{\alpha}D(f||f_u) \tag{2}$$

$$^{\alpha}\mathrm{RE} = \log n - \frac{1}{\alpha-1}\log\left[\left(\frac{1}{n}\right)^{1-\alpha}\left(\sum_{i=1}^{n} f_i^{\alpha}\right)\right] \tag{3}$$

$$^{\alpha}\mathrm{RE} = \log n - \left[\log n + \log\left(\sum_{i=1}^{n} f_i^{\alpha}\right)^{\frac{1}{\alpha-1}}\right] \tag{4}$$

$$\exp(^{\alpha}\mathrm{RE}) = \exp\left[\log\left(n \times \frac{(\sum_{i=1}^{n} f_i^{\alpha})^{\frac{1}{1-\alpha}}}{n}\right)\right] \tag{5}$$

$$^{\alpha}D = \mathrm{SR} \times \frac{^{\alpha}D}{\mathrm{SR}} \tag{6}$$

$$^{\alpha}D = \mathrm{SR} \times {}^{\alpha}E \tag{7}$$

□
