# Supplementary material for: A bioinformatic framework for immune repertoire diversity profiling enables detection of immunological status
Source: Genome Med. 2015 May 28;7(1):49. doi: 10.1186/s13073-015-0169-8 (PMC4489130; doi:10.1186/s13073-015-0169-8)
Supplement: Additional file 8: — Diversity and Evenness profiles of dataset 3 (BCR, baseline versus influenza vaccination) do not cluster by sampling time point but cluster apart from cluster profiles of dataset 2 (BCR, healthy versus CLL). a Hierarchical clustering of Diversity profiles of dataset 2 (healthy versus CLL) and dataset 3 (baseline versus influenza vaccination) was performed based on Euclidean distance and visualized using heatmaps. The heatmap depicts the pairwise distance coefficients of all profiles determined (see Methods for further details). Row colors describe dataset origin (dataset 2, red; dataset 3, blue), whereas column colors describe the different immunological statuses contained in datasets 2 (healthy, blue; CLL, red) and 3 (baseline, green; day 7, brown; day 21, violet). b Analogous analyses to (a) using Evenness profiles and correlation distance were performed. [file 13073_2015_169_MOESM8_ESM.pdf]

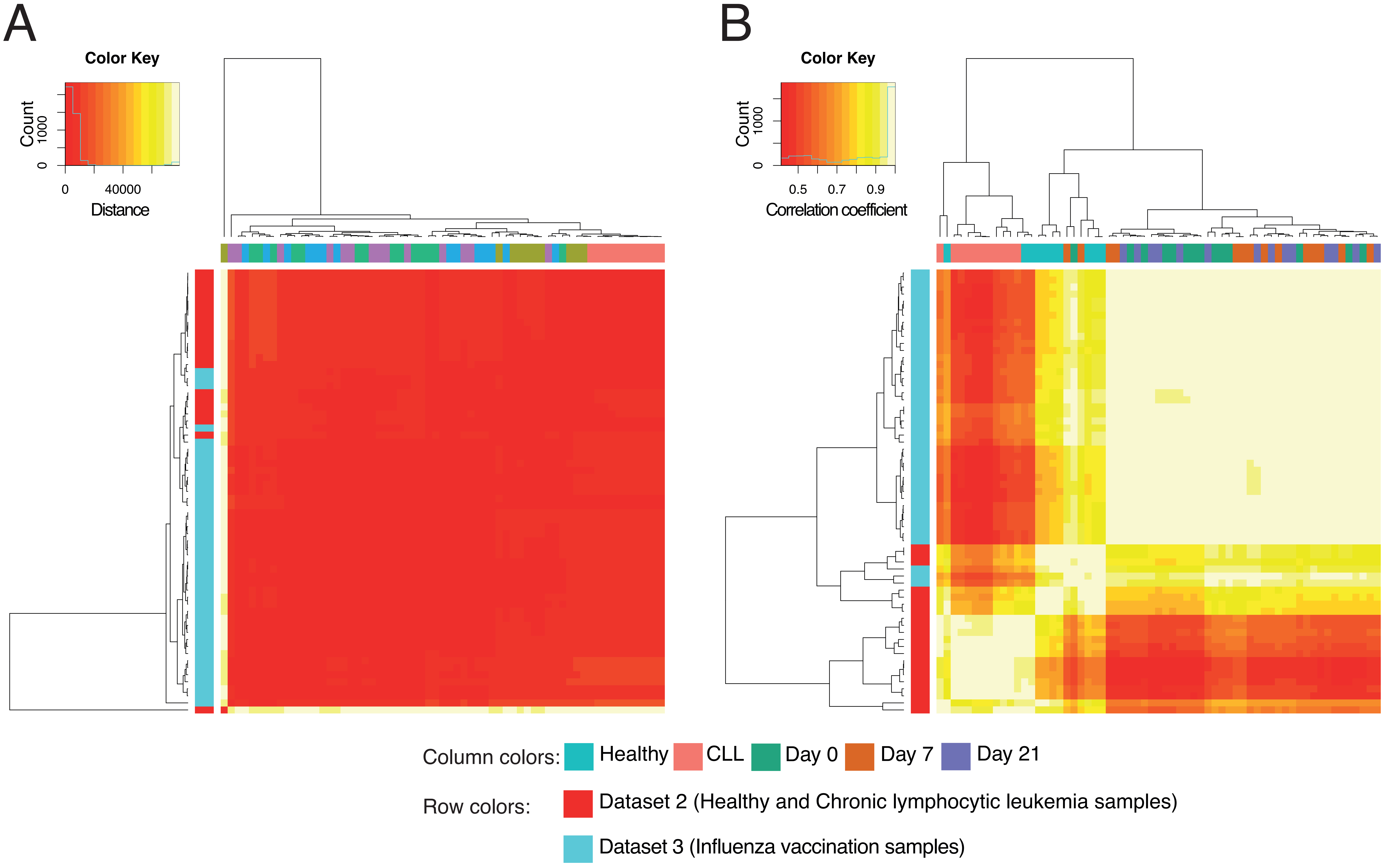
A
Color Key
Count
0
1000
0
40000
Distance
B
Color Key
Count
0
1000
0.5
0.7
0.9
Correlation coefficient
Column colors:
Healthy
CLL
Day 0
Day 7
Day 21
Row colors:
Dataset 2 (Healthy and Chronic lymphocytic leukemia samples)
Dataset 3 (Influenza vaccination samples)
