# Supplementary material for: A bioinformatic framework for immune repertoire diversity profiling enables detection of immunological status
Source: Genome Med. 2015 May 28;7(1):49. doi: 10.1186/s13073-015-0169-8 (PMC4489130; doi:10.1186/s13073-015-0169-8)
Supplement: Additional file 11: — Diversity ( α=1 D ) and Evenness ( α=1 E ) Shannon values classify TCR (Dataset 1) and BCR (Dataset 2) immune repertoires with lower prediction accuracy (BACC, >64 %) than the respective profiles (Table 1 ). BACCs were computed using nested leave-one-out cross-validation and were regarded as significant if p < 0.01. Legend: BACC (Sensitivity + Specificity)/2, balanced prediction accuracy. Please refer to Materials and Methods for more details. [file 13073_2015_169_MOESM11_ESM.docx]

Additional file 9

| **Dataset 1** | | | | | |
| --- | --- | --- | --- | --- | --- |
| Classification problem | BACC [%] | Sensitivity [%] | Specificity [%] | Significance (p-value) | Median number of alpha values used |
| CD4-Diversity (${}^{\alpha=1}D$):  Month 2 vs.  Baseline + Month 12 | 81.3 | 62.5 | 100 | 0 | 1 (alpha = 1) |
| CD4-Evenness (${}^{\alpha=1}E$):  Month 2 vs.  Baseline + Month 12 | 82.3 | 64.6 | 100 | 0 | 1 (alpha = 1) |
| CD8-Diversity (${}^{\alpha=1}D$):  Month 2 vs.  Baseline + Month 12 | 71.9 | 43.8 | 100 | 0.031 | 1 (alpha = 1) |
| CD8-Evenness (${}^{\alpha=1}E$):  Month 2 vs.  Baseline + Month 12 | 64.2 | 45.8 | 82.6 | 0.030 | 1 (alpha = 1) |
| **Dataset 2** | | | | | |
| Diversity (${}^{\alpha=1}D$):  Healthy vs. CLL | 84.6 | 69.2 | 100 | 0.015 | 1 (alpha = 1) |
| Evenness (${}^{\alpha=1}E$):  Healthy vs. CLL | 88 | 76 | 100 | 0.017 | 1 (alpha = 1) |
