# Supplementary material for: A bioinformatic framework for immune repertoire diversity profiling enables detection of immunological status
Source: Genome Med. 2015 May 28;7(1):49. doi: 10.1186/s13073-015-0169-8 (PMC4489130; doi:10.1186/s13073-015-0169-8)
Supplement: Additional file 12: — Diversity ( α=2 D ) and Evenness ( α=2 E ) Simpson-Index values classify TCR (dataset 1) and BCR (dataset 2) immune repertoires with lower BACC (prediction accuracy, >64 %) than the respective profiles (Additional file 11 ). BACCs were computed using nested leave-one-out cross-validation and were regarded as significant if p < 0.01. BACC (Sensitivity + Specificity)/2, balanced prediction accuracy. Please refer to Methods for more details. [file 13073_2015_169_MOESM12_ESM.docx]

Additional file 10

| **Dataset 1** | | | | | |
| --- | --- | --- | --- | --- | --- |
| Classification problem | BACC [%] | Sensitivity [%] | Specificity [%] | Significance (p-value) | Median number of alpha values used |
| CD4-Diversity (${}^{\alpha=2}D$):  Month 2 vs.  Baseline + Month 12 | 77.1 | 54.2 | 100 | 0 | 1 (alpha = 2) |
| CD4-Evenness (${}^{\alpha=2}E$):  Month 2 vs.  Baseline + Month 12 | 75.9 | 60.5 | 91.3 | 0 | 1 (alpha = 2) |
| CD8-Diversity (${}^{\alpha=2}D$):  Month 2 vs.  Baseline + Month 12 | 68.8 | 37.5 | 100 | 0.035 | 1 (alpha = 2) |
| CD8-Evenness (${}^{\alpha=2}E$):  Month 2 vs.  Baseline + Month 12 | 84 | 83.3 | 82.6 | 0 | 1 (alpha = 2) |
| **Dataset 2** | | | | | |
| Diversity (${}^{\alpha=2}D$):  Healthy vs. CLL | 76.9 | 53.8 | 100 | 0.011 | 1 (alpha = 2) |
| Evenness (${}^{\alpha=2}E$):  Healthy vs. CLL | 72.4 | 53.9 | 90.9 | 0.079 | 1 (alpha = 2) |

Diversity (${}^{\alpha=2}D$) and Evenness (${}^{\alpha=2}E$) *Simpson-Index values* classify TCR (Dataset 1) and BCR (Dataset 2) immune repertoires with lower BACC (prediction accuracy, >64%) than the respective profiles (Table S1). BACCs were computed using nested leave-one-out cross-validation and were regarded as significant if p<0.01. Legend: BACC (Sensitivity + Specificity)/2), balanced prediction accuracy. Please refer to *Materials and Methods* for more details.
